# Allosteric coupling of substrate binding and proton translocation in MmpL3 transporter from *Mycobacterium tuberculosis*

**DOI:** 10.1128/mbio.02183-24

**Published:** 2024-08-30

**Authors:** Svitlana Babii, Wei Li, Lixinhao Yang, Anna E. Grzegorzewicz, Mary Jackson, James C. Gumbart, Helen I. Zgurskaya

**Affiliations:** 1 Department of Chemistry and Biochemistry, University of Oklahoma, Norman, Oklahoma, USA; 2 Mycobacteria Research Laboratories, Department of Microbiology, Immunology and Pathology, Colorado State University, Fort Collins, Colorado, USA; 3 School of Chemistry and Biochemistry, Georgia Institute of Technology, Atlanta, Georgia, USA; 4 School of Physics, Georgia Institute of Technology, Atlanta, Georgia, USA; Case Western Reserve University School of Medicine, Cleveland, Ohio, USA

**Keywords:** *Mycobacterium tuberculosis*, outer membrane, trehalose monomycolate transport, proteoliposomes, lipid transport and inhibition

## Abstract

Infections caused by *Mycobacterium* spp. are very challenging to treat, and multidrug-resistant strains rapidly spread in human populations. Major contributing factors include the unique physiological features of these bacteria, drug efflux, and the low permeability barrier of their outer membrane. Here, we focus on MmpL3 from *Mycobacterium tuberculosis*, an essential inner membrane transporter of the resistance–nodulation–division superfamily required for the translocation of mycolic acids in the form of trehalose monomycolates (TMM) from the cytoplasm or plasma membrane to the periplasm or outer membrane. The MmpL3-dependent transport of TMM is essential for the growth of *M. tuberculosis in vitro*, inside macrophages, and in *M. tuberculosis-*infected mice. MmpL3 is also a validated target for several recently identified anti-mycobacterial agents. In this study, we reconstituted the lipid transport activity of the purified MmpL3 using a two-lipid vesicle system and established the ability of MmpL3 to actively extract phospholipids from the outer leaflet of a lipid bilayer. In contrast, we found that MmpL3 lacks the ability to translocate the same phospholipid substrate across the plasma membrane indicating that it is not an energy-dependent flippase. The lipid extraction activity was modulated by substitutions in critical charged and polar residues of the periplasmic substrate-binding pocket of MmpL3, coupled to the proton transfer activity of MmpL3 and inhibited by a small molecule inhibitor SQ109. Based on the results, we propose a mechanism of allosteric coupling wherein substrate translocation by MmpL3 is coupled to the energy provided by the downhill transfer of protons. The reconstituted activities will facilitate understanding the mechanism of MmpL3-dependent transport of lipids and the discovery of new therapeutic options for *Mycobacterium* spp. infections.

**IMPORTANCE:**

MmpL3 from *Mycobacterium tuberculosis* is an essential transporter involved in the assembly of the mycobacterial outer membrane. It is also an important target in undergoing efforts to discover new anti-tuberculosis drugs effective against multidrug-resistant strains spreading in human populations. The recent breakthrough structural studies uncovered features of MmpL3 that suggested a possible lipid transport mechanism. In this study, we reconstituted and characterized the lipid transport activity of MmpL3 and demonstrated that this activity is blocked by MmpL3 inhibitors and substrate mimics. We further uncovered the mechanism of how the binding of a substrate in the periplasmic domain is communicated to the transmembrane proton relay of MmpL3. The uncovered mechanism and the developed assays provide new opportunities for mechanistic analyses of MmpL3 function and its inhibition.

## INTRODUCTION

We live in the era of anti-microbial resistance that has spread in clinics and human populations and threatens our well-being and modern medical advances. Infections caused by *Mycobacterium tuberculosis* (*Mtb*) and related bacterial pathogens are notoriously difficult to treat with antibiotics. Intrinsic and acquired multidrug resistance of these pathogens demands new fundamental insights into underlying molecular mechanisms that will facilitate the discovery of new therapeutics. One of the major mechanisms protecting mycobacteria from the action of antibiotics is the low permeability barrier of the mycobacterial outer membrane (mycomembrane). The inner leaflet of mycomembranes is formed by mycolic acids (C60-C90 carbons in lengths) covalently attached to the underlying arabinogalactan, whereas the outer leaflet is composed of a diversity of both conserved and species-specific, non-covalently attached lipids, glycolipids, and lipoglycans. The assembly of mycomembranes requires functions of MmpL efflux transporters from the resistance–nodulation–division (RND) superfamily of proteins.

This study is focused on the biochemical mechanism of MmpL3, which transports trehalose monomycolates (TMM) and is a validated target for several recently identified anti-mycobacterial agents (1–3). Genetic or chemical inhibition of MmpL3 activity *in vivo* reduces cell envelope mycolylation (4, 5). Mycolic acids are synthesized in the cytoplasm and esterified to the disaccharide trehalose to generate TMM (3). TMM then serves as a mycolic acid donor in the formation of mycolic acid-containing lipids (e.g., trehalose dimycolate [TDM]) found in the outer leaflet of the mycomembrane, as well as the cell wall-bound mycolates, which constitute the inner leaflet of the mycomembrane (6). The MmpL3-dependent transport of TMM is essential for the growth of *Mtb in vitro*, inside macrophages, and in *Mtb-*infected mice (4, 7, 8).

Indirect evidence based on X-ray crystallography and other biophysical approaches suggests that MmpL3 might carry additional substrates in addition to TMM. The X-ray structure of MmpL3 revealed the presence of the major phospholipid phosphatidylethanolamine (PE) bound in the substrate-binding site of MmpL3 (9). The bound PE was observed in the whole-length MmpL3 purified not only from *Escherichia coli* but also from *Mycobacterium smegmatis* (*Msmg*). In addition, both phosphatidylglycerol and cardiolipin were found to specifically interact with MmpL3, suggesting that all these phospholipids are potential substrates for MmpL3. The PE translocation was also observed during molecular dynamics (MD) simulations (10).

The structure of MmpL3 is representative of other RND transporters and comprises 12 transmembrane α-helices (TMs), two large periplasmic loops between TM1 and TM2 (PN, *Mtb* MmpL3 residues 37–166) and TM7 and TM8 (PC, *Mtb* MmpL3 residues 415–544), and a cytoplasmic domain (9, 11, 12). The two periplasmic loops form a large central cavity, which is a binding site of TMM analogs, PE, and detergents. The C-terminal cytoplasmic domain is unstructured in the purified MmpL3; it is not essential for its transport function but is the site of interaction with various protein partners and promotes trimerization of MmpL3 (13). As with other RND transporters, MmpL3 activity is driven by the proton-motive force. Two sets of Asp-Tyr dyads located in the TM4 and TM10 of MmpL3 are thought to form the proton transfer relay, which is also the site of binding and action of several structurally diverse inhibitors of MmpL3 such as SQ109, indolecarboxamides, and BM212 (12). The H^+^ transfer activity of MmpL3 and its modulation by inhibitors have been validated *in vitro* with the purified MmpL3 reconstituted into proteoliposomes (PLs) (10).

Recent structural and computational studies suggested that MmpL3 binds its lipid substrates from the outer leaflet of the cytoplasmic membrane and translocates them through a channel (11, 14). The substrate is oriented perpendicular to the plane of the cytoplasmic membrane by hydrophobic packing of the fatty acid tail with the hydrocarbon chains and trehalose stabilization by phosphoryl moieties of phospholipids. A groove surrounded by TM7–10 forms the entrance of the translocation channel, which spans the outer leaflet of the cytoplasmic membrane and up to the central cavity located in the periplasmic domain of MmpL3. In the proposed mechanism, substrates are shuttled through the channel to reach the periplasmic binding pocket (PBP). At least five different ligands were resolved in the PBPs of *Msmg* and *Mtb* MmpL3: (i) 6-n-dodecyl-α,α-trehalose (6DDTre), a structural analog of TMM (12); (ii) detergent dodecyl maltoside (DDM) (12); (iii) PE (9); (iv) lauryl maltose neopentyl glycol (15); and (v) TMM (11) (Fig. S1). All these ligands have striking similarities in their binding poses in the PBP, in which interactions with the alkyl chains are separated in the PBP from the headgroups of lipids (15). The central vestibule sequesters the alkyl chains away from the periplasm, while the proximate hydrophilic openings bind the polar head groups through hydrogen (H) bonds and electrostatic interactions providing it a degree of solvent access. The alkyls flip along the hydrophobic surface of the PBP vestibule while the headgroups are tightly held by the H-bonds. However, biochemical validation of this mechanism is missing, and it remains unclear whether MmpL3 can flip lipids across the membrane and/or extract lipids from the outer leaflet of the membrane. Since the PBP is separated from the H^+^ transfer relay, how the transport of lipids is coupled with H^+^ transfer is also unknown.

In this study, we reconstituted the lipid transfer activity of the purified MmpL3 using a two-lipid vesicle system and established for the first time the ability of MmpL3 to actively extract phospholipids from the outer leaflet of a lipid bilayer. MmpL3, however, fails to act as an energy-dependent flippase with the same phospholipid substrate. The lipid extraction activity was modulated by substitutions in the critical charged and polar residues in the PBP of MmpL3, coupled to the H^+^ transfer activity of MmpL3 and inhibited by the addition of TMM analogs and a small molecule inhibitor.

## RESULTS

### MmpL3 reconstituted into proteoliposomes expels lipids from a lipid bilayer

**Fig 1:**
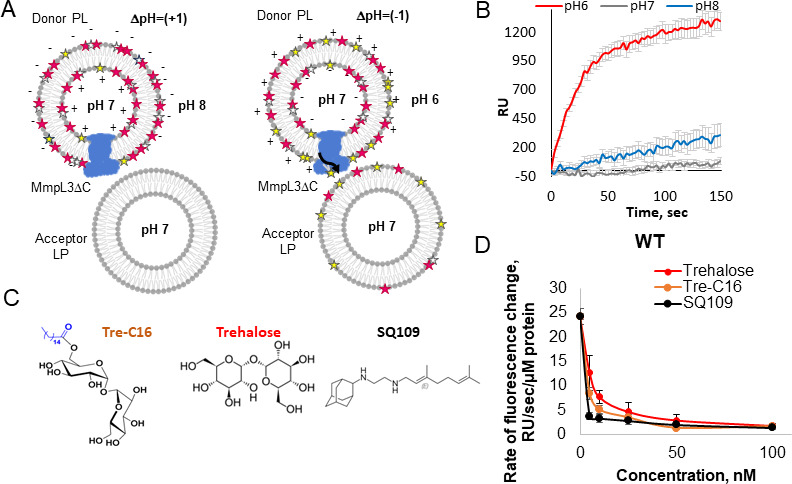
Intermembrane lipid transport activity of MmpL3ΔC. (**A**) A scheme of two-lipid vesicle assay. Donor PLs containing MmpL3ΔC transporter and protein-free control vesicles (control) were mixed with a large excess of acceptor vesicles, and NBD fluorescence was monitored at 30°C (relative units). The ΔpH was generated by diluting the vesicles (internal pH, 7.0) into buffer of pH 6.0 or pH 8.0. The transport reaction was initiated by the addition of 5 mM MgCl_2_ and terminated by the addition of EDTA (10 mM). (**B**) Kinetics of lipid transport by MmpL3ΔC at different external pH. Fluorescence changes of MmpL3∆C PLs in the external buffers with pH 6.0 (red line), pH 7.0 (gray line), and pH 8.0 (blue line) were monitored at λ_ex_ = 460 nm and λ_em_ = 536 nm for 2.5 min at 30°C. All plots were normalized to zero-time initial fluorescence. Error bars are standard errors (SE) (*n* = 9). (**C**) Chemical structures of a TMM analog Tre-C16, trehalose, and an inhibitor SQ109 used in this study. (**D**) Rates of intermembrane lipid transfer by MmpL3 at ΔpH = (−1) and increasing concentrations of substrate analogs and SQ109 inhibitor. Error bars are SE (*n* = 9).

To study lipid transport, we reconstituted MmpL3 into proteoliposomes containing fluorescently labeled PE (16). In this intermembrane lipid transport assay, the purified *Mtb* MmpL3 lacking the C-terminal cytoplasmic domain (MmpL3ΔC) (10) was reconstituted into proteoliposomes containing fluorescent N-7-nitrobenz-2-oxa-1,3-diazol-4-yl (NBD)- and N-lissamine rhodamine B sulfonyl (Rh)-labeled PE (Fig. S2). The fluorescent lipids are incorporated into MmpL3ΔC proteoliposomes at concentrations sufficient for the quenching of NBD fluorescence due to fluorescence energy transfer (FRET) to Rh (0.5% of total lipids) (Fig. 1A). The transfer of fluorescently labeled PE from the MmpL3 proteoliposomes into acceptor protein-free lipid vesicles devoid of fluorescently labeled PE is initiated by imposing the basic inside ΔpH that will drive the extraction of lipids by MmpL3 from the outer leaflet of the lipid bilayer and by the addition of MgCl_2_ to bring the donor and acceptor vesicles into contact (Fig. 1A). In the presence of an excess of acceptor liposomes, the transport of fluorescent lipids by MmpL3ΔC will decrease the concentration of fluorescent lipids in proteoliposomes and, hence, increase the distance between the NBD- and Rh-labeled phospholipids. The lipid transfer can be monitored in real time by following the increase in the NBD fluorescence (Fig. 1B).

We found that the reconstituted MmpL3ΔC effectively transported labeled PE from donor proteoliposomes into the acceptor vesicles, as seen from the increased NBD fluorescence, which reached the steady state at ~1 min after the reaction was initiated (Fig. 1B). This transport was highly efficient when the MmpL3ΔC proteoliposomes and donor vesicles were reconstituted in pH 7.0 and the transport reaction was carried out in the external buffer pH 6.0 [ΔpH = (−1)] (Fig. 1B). However, slow spontaneous transfer of labeled PE could also be seen when ΔpH = 1 was imposed by carrying out the reaction in the external buffer pH 8.0 and in the absence of energy at ΔpH = 0. At ΔpH = 1, the proton-motive force is directed from inside the proteoliposomes out and cannot drive the energy-dependent intermembrane efflux of lipids. Thus, some lipid transfer was occurring in an energy-independent manner.

The previously measured affinity of TMM (3.7 ± 1.3 µM) to MmpL3 was higher than that of PE (19.5 ± 6.3 µM) (9). We next tested whether the TMM analog Tre-C16 (Fig. 1C), containing a trehalose headgroup acylated with a 16-carbon-long fatty acid tail (C16) (10), can compete with NBD-PE and/or Rh-PE for the binding to MmpL3ΔC. We found that Tre-C16 efficiently inhibited the PE efflux by MmpL3ΔC with the half-inhibitory concentration (IC_50_) = 6.5 ± 0.6 nM (Fig. 1D). Interestingly, free L-trehalose also inhibited the lipid transfer activity, but the IC_50_ was higher (11.4 ± 1.3 nM) (Fig. 1D). Finally, inhibitor SQ109, which binds in the proton transfer site of MmpL3 formed by amino acid residues in TM4 and TM10, also inhibited the lipid transfer with the lowest IC_50_ value of 4.95 ± 0.2 nM (Fig. 1D).

Thus, MmpL3ΔC purified and reconstituted into proteoliposomes transports fluorescently labeled phospholipids. The Tre-C16 and free trehalose inhibit this activity presumably by competing with phospholipids for binding to the transporter. SQ109 also inhibit this MmpL3ΔC activity by binding to the proton relay residues and inhibiting the proton transfer activity of MmpL3ΔC.

### Amino acid substitutions in the substrate-binding site of MmpL3 diminish its activity in whole cells

**Fig 2:**
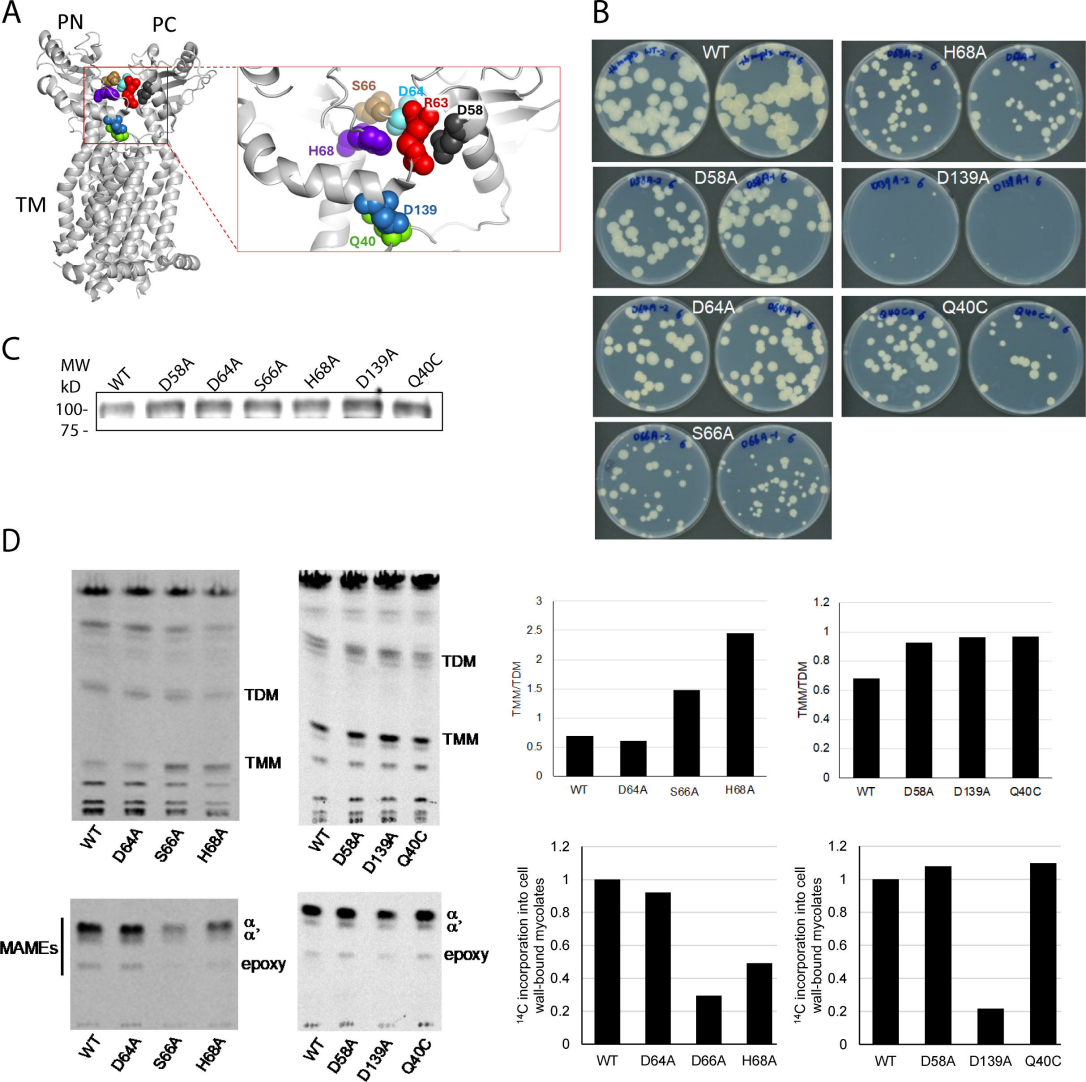
Effect of amino acid substitutions on the activity of MmpL3. (**A**) Structure of *Mtb* MmpL3 (PDB ID: 7NVH) with residues targeted for mutagenesis shown as spheres. The inset shows the PBP with mutated residues indicated. (**B**) Growth of *Msmg*Δ*mmpL3* expressing either a wild-type (WT) copy of *mmpL3* from *M. tuberculosis* or mutated variants of this gene on 7H11-ADC agar plate containing 25 µg/mL kanamycin and 50 µg/mL hygromycin and incubated at 37°C for 5 days. (**C**) Production of *Mtb* MmpL3 and mutated variants by the recombinant strains. Twenty-five micrograms of total protein extracts from each of the strains shown in panel **A** was separated by SDS-PAGE and analyzed by immunoblot using a polyclonal rabbit antibody directed against the first periplasmic loop of MmpL3tb. (**D**) Total [1,2-^14^C]acetate-derived lipids and cell wall-bound mycolic acids from the recombinant strains were analyzed by thin-layer chromatography (TLC) in the solvent systems CHCl_3_/CH_3_OH/H_2_O (20:4:0.5, by vol.) for total lipids and *n*-hexane(s)/ethyl acetate (95:5, by vol.; two developments) for mycolic acid methyl esters (MAMEs). For lipid analysis, the same total counts (10,000 dpm) for each sample were loaded per lane. The amount of radioactivity incorporated into TMM and TDM was semi-quantified using a Sapphire Biomolecular Imager, and the results, expressed as TMM/TDM ratios, are presented alongside the autoradiograms. For cell wall-bound mycolic acid analysis, the same volume of samples was loaded for each strain, and the amount of radioactivity incorporated into all forms of MAMEs (alpha, alpha prime, and epoxy-mycolates) was semi-quantified as above. Radioactivity incorporation in the cell wall-bound mycolic acids of the mutants is expressed relative to that incorporated by the strain expressing a WT version of *mmpL3* (arbitrarily set to 1). Reduced *Mtb* MmpL3 activity in some of the mutants results in a decrease in mycolic acid transfer to arabinogalactan and reduced incorporation into TDM concomitant with an accumulation of TMM. The metabolic labeling results presented are representative of two to three metabolic labeling experiments conducted on two independent clones of each *Mtb* MmpL3 mutant.

The *in vitro* experiments described above suggested that the MmpL3 residues involved in the interactions with the trehalose moiety are important for lipid transport activity. MD simulations of MmpL3 interactions with TMM identified D58, D64, S66, H68, and D139 in the PBP among amino acid residues H-bonding with the head group of TMM (14) (Fig. 2A). The side chains of D58 and D139 were also found to form H-bonds with the trehalose head group of 6-n-dodecyl-α,α-trehalose detergent in the crystal structure of MmpL3 from *Msmg* (12) and the maltose head group of lauryl maltose neopentyl glycol in the cryo-electron microscopy(EM) structure of *Mtb* MmpL3 (15) (Fig. S1). In addition, the side chains of R63 and Q40 of MmpL3 are also engaged in electrostatic and polar interactions with the trehalose head group of the substrate in the cryo-EM analyses (11). We next substituted Ala for each of the polar residues D58, R63, D64, S66, H68, and D139 in *Mtb* MmpL3 and tested the ability of these variants expressed under the control of the *hsp60* promoter from the replicative plasmid pMVGH1 to rescue the growth of a *Msmg*Δ*mmpL3* knockout mutant as described previously (17). We similarly analyzed the Q40C mutant reported earlier (17).

We found that out of seven mutated *Mtb* MmpL3 variants, six were able to support the growth of *Msmg*Δ*mmpL3* indicating that they retained at least partial functionality (Fig. 2). The R63A mutant had a severe growth deficiency and failed to grow after reinoculation. Among the remaining six mutants, four had reduced growth rates at 37°C (Fig. 2B) with MmpL3^D139A^, MmpL3^S66A^, and, to a lesser extent, MmpL^H68A^ and MmpL3^Q40C^ consistently displaying the most pronounced phenotypes. The fact that MmpL3 variants were expressed at similar levels in *Msmg*Δ*mmpL3* was confirmed by immunoblot using anti-MmpL3 antibodies (Fig. 2C).

MmpL3 activity in the different strains was next assessed and compared by monitoring the incorporation of [1,2-^14^C]acetate into their arabinogalactan-bound mycolates, TMM and TDM. In line with their reduced growth rate, the strains producing MmpL3^D139A^, MmpL3^S66A^, and MmpL^H68A^ presented reduced rates of mycolic acid transfer to their cell envelope acceptors, arabinogalactan and/or TDM, indicative of reduced MmpL3 activity (Fig. 2D). Thus, substitutions in the PBP of MmpL3 have a detrimental effect on bacterial growth due to defects in mycolic acid transfer to their cell envelope acceptors.

### Mutations in the substrate-binding site of MmpL3ΔC modulate both the reconstituted lipid and proton transfer activities

**Fig 3:**
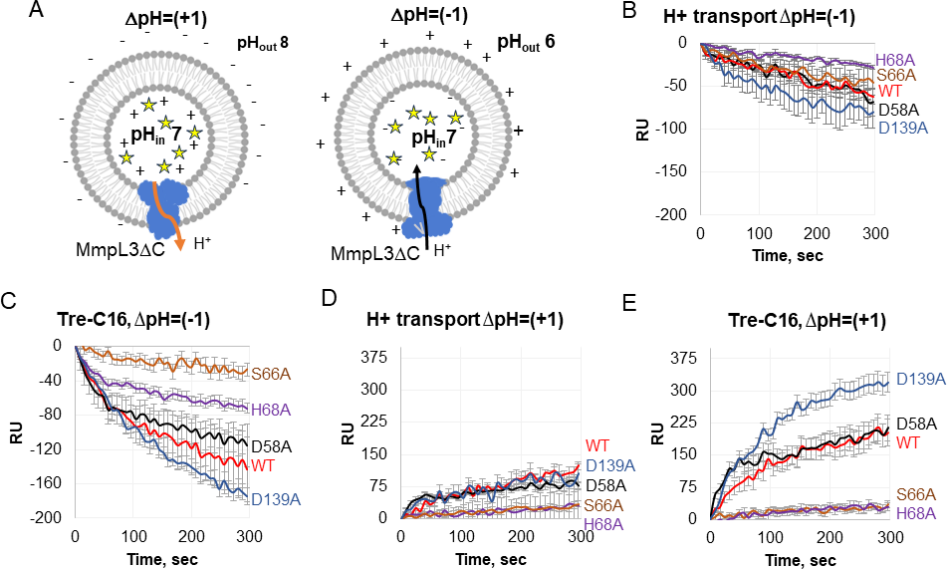
Proton transfer activity of MmpL3ΔC variants. (**A**) A scheme of H^+^ transfer assay. Proteins are reconstituted into proteoliposomes and loaded with the pH-sensitive fluorescent probe pyranine in a buffer of pH 7.0. The ΔpH is generated by diluting PLs either into the buffer of pH 6.0 or pH 8.0. The fluorescence of pyranine decreases upon protonation in acidic pH and increases upon deprotonation. (**B**) H^+^ transport activities of indicated MmpL3ΔC variants with imposed ΔpH = (−1). Fluorescence of pyranine (λ_ex_ = 455 nm and λ_em_ = 509 nm) was monitored for 5 min at 25°C. (**C**) The same as panel B but in the presence of 50 nM Tre-C16 substrate analog. (**D**) H^+^ transport activities of indicated MmpL3ΔC variants with imposed ΔpH = (+1). (**E**) The same as panel D but in the presence of 50 nM Tre-C16 substrate analog. All plots were normalized to zero-time initial fluorescence. Error bars are SE (*n* = 9).

We next analyzed how mutations in the PBP of MmpL3tb affect the lipid and H^+^ transfer activity of the transporter. For this purpose, we constructed MmpL3ΔC variants carrying D58A, S66A, H68A, and D139A substitutions in the PBP (Fig. 2), purified these protein variants, and reconstituted them into proteoliposomes (Fig. S2 to S4). The R63 variant was also constructed, but the purified protein was unstable in detergent solutions, suggesting folding defects, and was omitted from further studies. The lipid transport activity of the proteins reconstituted into proteoliposomes was analyzed as described above (Fig. 1). To monitor the H^+^ transfer activity, the proteoliposomes were loaded with the pH-sensitive probe pyranine, the fluorescence of which increases when pH inside the proteoliposomes increases or decreases during the acidification of the proteoliposome lumen (Fig. 3A). In agreement with the previous studies (10), the reconstituted MmpL3ΔC was found to translocate protons in both directions at ΔpH = (−1) and ΔpH = 1, but the H^+^ transfer was more efficient at ΔpH = (−1) (Fig. 3B and D; Table S1). This activity was strongly stimulated by Tre-C16 (Fig. 3C and E) and weakly by free trehalose (Fig. S3).

**Fig 4:**
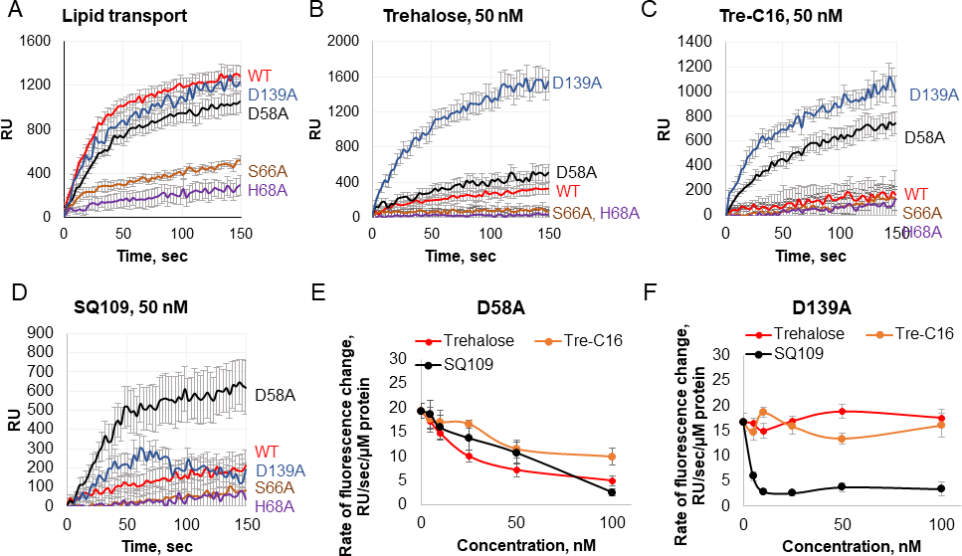
Intermembrane lipid transport activity of MmpL3ΔC variants and the effect of substrate analogs and SQ109. (**A**) Kinetics of lipid transport by MmpL3ΔC WT and indicated mutant variants at the external pH 6.0. Fluorescence changes were monitored at λ_ex_ = 460 nm and λ_em_ = 536 nm for 2.5 min at 30°C. (**B–D**) The same as panel A but in the presence of 50 nM trehalose (**B**), Tre-C16 (**C**), and SQ109 (**D**). (**E and F**) The effect of increasing concentrations of the indicated substrate analogs and SQ109 on the rates of intermembrane lipid transport by D58A (**E**) and D139A (**F**) variants. Error bars are SE (*n* = 9).

In agreement with the whole-cell assays that identified the D58A variant as the most active among the constructed *Mtb* MmpL3 mutants (Fig. 2), we found that the lipid and H^+^ transfer activities of MmpL3ΔC^D58A^ were like those of the parent MmpL3ΔC protein (Fig. 3B and C and 4A; Fig. S4). Similarly, the H^+^ transfer activity of MmpL3ΔC^D58A^ was also stimulated by Tre-C16 (Fig. 3C) and weakly by free trehalose but only at ΔpH= (−1) (Fig. S3). Surprisingly, unlike with the parent MmpL3ΔC protein, the fluorescent lipid transport by MmpL3ΔC^D58A^ was only weakly inhibited by Tre-C16 or free trehalose with IC_50_ values of 83.6 ± 13.9 and 29.3 ± 4.1 nM, respectively (Fig. 4B and C; Table S2). Thus, this mutant became more specific to PE and interacted less efficiently with other ligands.

**Fig 5:**
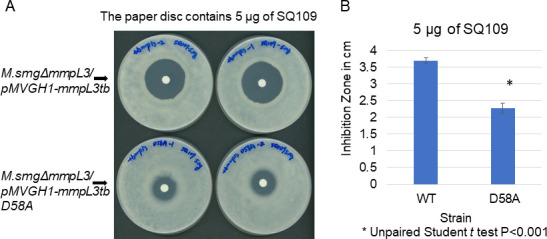
Effect of D58A substitution in *Mtb* MmpL3 on the susceptibility of *M. smegmatis* to SQ109 inhibitor. (**A**) Disc diffusion assay. Zones of inhibition of SQ109-containing discs on 7H11-ADC agar plates seeded with ~10^6^ cells of MsmgDmmpL3/pMVGH1-mmpL3tb (WT) and MsmgΔmmpL3/pMVGH1-mmpL3tb (D58A). Plates were incubated for 4 days at 37°C. (**B**) Measured sizes of the zones of inhibition (in centimeters) for discs containing 5 µg of SQ109 (means standard deviations of two independent clones for each strain).

Surprisingly, SQ109 only partially inhibited the lipid transport by MmpL3ΔC^D58A^ (IC_50_ = 30.1 ± 7.7 nM) (Fig. 4D and F), pointing to the disruption of coupling between the substrate and H^+^ transport reactions in this mutant protein. In agreement, we found that *Msmg* cells producing *Mtb* MmpL3 D58A mutant were less susceptible to growth inhibition by SQ109 than cells producing the wild-type (WT) *Mtb* MmpL3. As seen from the results of the disc diffusion assay, the zone of inhibition by SQ109 is significantly larger on the lawn of cells carrying *Mtb* MmpL3 than on D58A mutant cells (Fig. 5). This result suggests that D58 contributes to communicating the ligand occupancy in the PBP to the H^+^ relay in the TM domain of MmpL3.

The biochemical activities of other mutants were dramatically different from those of the D58A variant. Both MmpL3ΔC^H68A^ and MmpL3ΔC^S66A^ variants were deficient in the lipid and H^+^ transfer activities (Fig. 3B and D and 4A; Fig. S4), suggesting that these amino acid substitutions disrupt the recognition and binding of ligands in MmpL3. MmpL3ΔC^S66A^ was more active than MmpL3ΔC^H68A^ in the lipid transport assay (Fig. 4A; Table S2), but the opposite was true for the H^+^ transport assay, in which MmpL3ΔC^H68A^ was somewhat more active than MmpL3ΔC^S66A^ (Fig. 3B and D). In addition, both mutants were somewhat more active in the H^+^ transport at ΔpH = (−1) than at ΔpH = 1. This result suggests that the positive charge on the imidazole moiety of H68 (pKa ~6.2) is important for the activities of both mutants. Both the trehalose and Tre-C16 ligands weakly stimulated MmpL3ΔC^H68A^ at ΔpH = (−1), but no significant stimulation was found for MmpL3ΔC^S66A^ (Fig. 4B and C). Interestingly, the remaining lipid transfer activity of MmpL3ΔC^S66A^ was inhibited by these ligands and SQ109 inhibitor (Fig. 4D; Fig. S5; Table S2). Thus, unlike with MmpL3ΔC^D58A^, diverse lipids can access the PBP of this mutant, and the mutation did not disrupt the communication between the PBP and the proton relay residues.

Finally, the MmpL3ΔC^D139A^ variant, which had the deepest defect in translocation of TMM in whole cells, was as efficient at H^+^ and lipid transport as the parent MmpL3ΔC protein (Fig. 3B, D and 4A; Table S2). The H^+^ transfer activity of MmpL3ΔC^D139A^ was stimulated by Tre-C16 even more strongly than the parent protein, especially at ΔpH = 1 (Fig. 3B and D). The lipid transport activity of this mutant protein, on the other hand, was not affected by substrate analogs, as seen from the lack of inhibition by Tre-C16 and trehalose, IC_50_ > 100 nM (Fig. 4B, C, and F). However, this mutant was still fully inhibited by SQ109 with IC_50_ = 6.0 ± 0.6 nM (Fig. 4D and F). Thus, similar to the D58A variant, the D139A variant fails to establish the same interactions with Tre-C16 and trehalose as the parent protein, but unlike D58A, its communication with the H^+^ transfer relay is preserved.

Taken together, our results show that mutations in the PBP of MmpL3 have a strong and residue-specific effect on the lipid and H^+^ transfer activity of the transporter. Since the PBP and the residues forming the proton relay are in the two different domains of MmpL3, this result suggests that the binding of ligands in the PBP is allosterically coupled to the conformational changes in the transmembrane domain of MmpL3.

### MmpL3 does not carry out an energy-dependent transport of phospholipids across a bilayer

**Fig 6:**
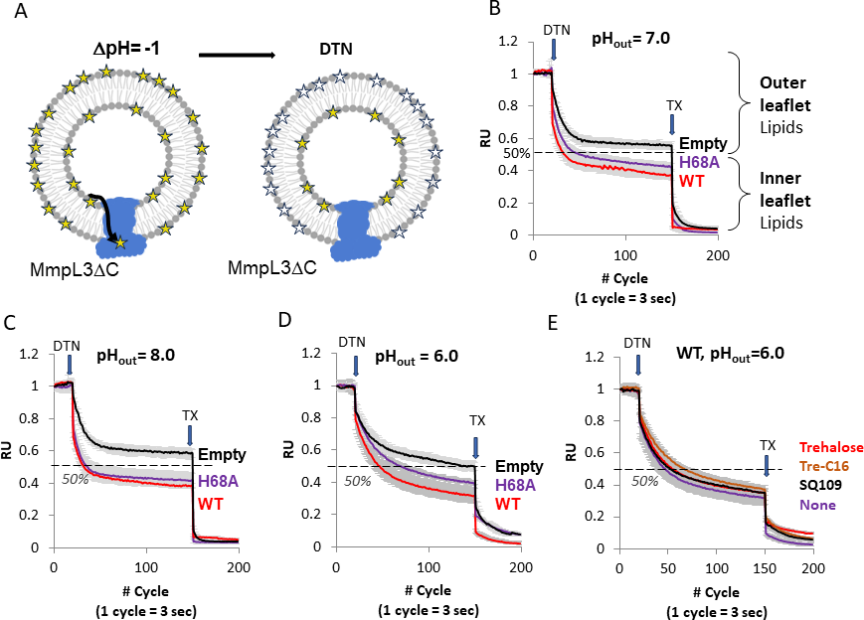
Flippase activities of MmpL3∆C and its H68A variant. (A) A scheme of flippase activity assays. MmpL3∆C WT and H68A proteins were reconstituted into PLs containing 0.5% of NBD-PE. DTN was added to quench the fluorescence of NBD-PE in the outer leaflet of lipid vesicles. Reaction was stopped after adding Triton X-100, which dissolves lipid bilayers allowing DTN to quench the remaining inner leaflet NBD-PE. (B–D) NBD-PE flippase activity measured in HEPES-KOH buffer pH 7.0 (B), HEPES-KOH buffer pH 8.0 (C), and MES-HCl buffer pH 6.0 (D). (E) Effect of 100 nM trehalose (red line), Tre-C16 (orange line), and SQ109 (black line) on flippase activity of MmpL3∆C. Error bars are SE (*n* = 9).

We also analyzed whether MmpL3 possesses a flippase activity and can transport lipids across a bilayer. In this assay, MmpL3ΔC was reconstituted into proteoliposomes containing NBD-labeled PE, and then, the transporter was energized by imposing a positive or a negative ΔpH across the phospholipid bilayer. In the absence of transport and in control protein-free liposomes, the distribution of the NBD-labeled PE is expected to be random between the two leaflets of a lipid bilayer (~50% in both leaflets) that can be determined by adding a membrane-impermeable reagent dithionite (DTN) (Fig. 6A). DTN will quench the NBD fluorescence in the outer leaflet of the lipid bilayer but not the inner leaflet.

Typically, the random distribution of NBD-PE between the outer and inner leaflets of lipid vesicles is 55%/45% due to the larger surface area of the outer leaflet (18). As expected, we found that the addition of DTN reduced the fluorescence of the control protein-free vesicles by ~50% (Fig. 6B; Table S1). This distribution was not affected by different pH_out_ values, which is consistent with previous findings that DTN does permeate the lipid bilayer in the pH 6.0–8.0 range (18), but some permeation and slower quenching reaction can be seen at pH_out_ = 6.0 (Fig. 6C and D). In MmpL3ΔC proteoliposomes, the distribution of lipids was significantly skewed toward the outer leaflet reaching ~60% at pH_out_ = 7.0–8.0 (Fig. 6B and C) and ~68% of NBD-lipids localized outside at pH_out_ = 6.0 (Fig. 6D). This result suggested that at least some of the asymmetry in the lipid distribution could be caused by the presence of MmpL3ΔC.

However, the asymmetry of lipid distribution in proteoliposomes was unaffected by the presence of substrate analogs trehalose and Tre-C16 as well as by the inhibitor SQ109 (Fig. 6E). Since SQ109 binds to the proton transfer site of MmpL3 and inhibits the proton transfer activity of the transporter, these results suggest that MmpL3ΔC-dependent asymmetry in proteoliposomes is not driven by an active transport mechanism.

To further analyze whether flipping of lipids by MmpL3ΔC requires H^+^ transfer and/or binding of lipids in the PBP, we reconstituted and analyzed the NBD-PE distribution in MmpL3ΔC^H68A^ proteoliposomes that are defective in the H^+^ transfer as well as the extraction of fluorescently labeled PE from the outer leaflet of the lipid bilayer (Fig. 3 and 4). We found no significant differences in the NBD-PE distribution in H68A- and WT-containing proteoliposomes at pH_out_ = 7.0–8.0. At pH_out_ = 6.0, the outer leaflet of MmpL3ΔC^H68A^ proteoliposomes contained ~8% less NBD-PE than the WT.

Thus, we conclude that MmpL3ΔC does not carry out an energy-dependent transport of phospholipids across a lipid bilayer but can facilitate spontaneous flipping of lipids from the inner to the outer leaflet of the membrane. This flipping does not require H^+^ transfer, but it is less pronounced with H68A mutant variants.

### Hydrogen and electrostatic bonding with the headgroups of ligands is critical for the activity of MmpL3

The substitutions in the PBP of MmpL3 led to notable defects in bacterial growth and TMM transport in whole cells (Fig. 2) and very distinct properties in the reconstituted transport assays (Fig. 3 and 4). These residues are located in three regions of MmpL3: (i) D58 is located at the C-terminus of α1 helix of the PN domain, which is inserted into the PC domain; (ii) R63, D64, S66, and H68 are in the flexible loop connecting the PN to the PC domain; and (iii) Q40 capping the C-terminal end of TM1 and D139 in the α4 helix of the PN domain are H-bonded and located at the interface between the PBP and the outer leaflet of the cytoplasmic membrane (Fig. 2A). The structural and MD studies further suggest that the polar headgroup of TMM could be pulled from the membrane into the PBP by affinity toward the polar region of PBP where it H-bonds with the targeted amino acid residues (9, 11, 12, 14, 15). We next analyzed the H-bonding networks in the PBP of *Mtb* MmpL3 residues in the apo state as well as bound to TMM. We simulated three replicas for WT MmpL3 with TMM in the PBP and one replica each of MmpL3 with mutations D139A, D58A, H68A, R63A, or S66A, also with TMM bound. Thus, in total, we ran eight simulations. We calculated the root mean square deviation (RMSD) over these simulations (Fig. S6A), finding that the fluctuations are broadly similar among all simulations.

Altering these residues significantly influenced how TMM interacted with the MmpL3 binding site. The clearest indication of this was seen in the number of H-bonds between the ligand and the binding site (Fig. S6B, top; Table 1). We observed that mutations generally did not change the number of H-bonds with TMM, but they do significantly decrease the number of H-bonds with the five amino acids focused on here (Fig. S6B, bottom). In the WT protein, D58 showed the highest H-bond occupancy followed by S66 and D139. The alanine substitutions for the S66A, R63A, D58A, and D139A mutants led to the loss of H-bonds between TMM and all five residues. However, H68A exhibited the opposite behavior, in which the number of H-bonds increased, especially with D139 (Fig. S6; Table 1).

**TABLE 1:** Occupancy of H-bonds formed between TMM and selected residues

Name	Residue no.				
	139	58	63	66	68
D139A-TMM	0.0%	0.0%	0.0%	14.0%	0.0%
D58A-TMM	0.0%	0.0%	0.0%	0.2%	67.9%
H68A-TMM	56.4%	21.8%	16.0%	3.8%	0.1%
R63A-TMM	0.0%	0.0%	0.0%	0.0%	6.3%
S66A-TMM	0.0%	0.0%	0.0%	0.4%	0.0%
WT-TMM (avg)	14.3%	40.5%	5.8%	23.0%	9.8%

**Fig 7:**
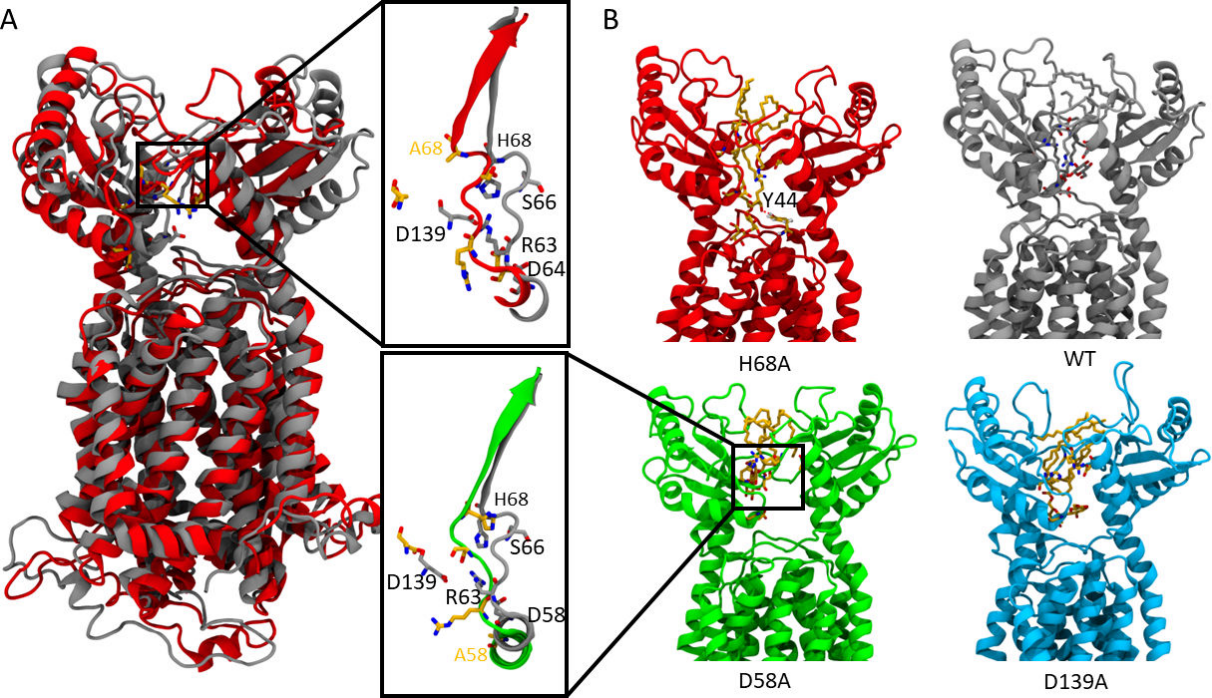
Simulations of MmpL3 with TMM bound. (**A**) Side view of *Mtb* MmpL3 H68A (red) and WT (gray). Panels on the right show secondary structure variations for the H68A (red) and D58A (green) mutants at the periplasmic binding site; WT is shown in gray for comparison. Five residues involved in the mutation simulations are displayed in licorice representation. (**B**) Snapshots of the most extreme TMM binding positions during simulations of H68A (red), WT (gray), D58A (green), and D139A (blue). For H68A, residue Y44 is also displayed in licorice representation, and the H-bond formed between Y44 and the TMM head group is indicated by a red dashed line.

Since the mutated residues directly bind TMM, their substitutions can potentially influence the overall binding pose of the substrate. To quantitatively analyze changes in binding positions, we next calculated the relative distances between the center of mass of the trehalose group of TMM and the center of mass of the MmpL3 protein (Fig. S7). Our analyses suggest that D58A binds TMM at a position closer to the periplasmic exit compared to WT, while other mutants bind it at a deeper position. Notably, TMM in H68A adopts a significantly deeper position (Fig. 7).

As described above, the D58A and H68A mutant exhibited several distinct behaviors. We next carried out a more detailed examination of these mutants when binding with TMM. The unique binding position of TMM in the D58A mutant is due to the flipping of the H68 residue (Fig. 7A). In the WT, the H68 residue points toward the membrane and forms H-bonds with the TMM head group beneath the loop. The D58A mutation causes the loop to alter its conformation, exposing H68 to TMM and allowing its side chain to point toward the center of the PD domain. This exposure facilitates a more stable H-bond between H68A and the TMM head group (Table 1), stabilizing TMM binding at a higher position.

Conversely, the H68A mutation results in a deeper TMM binding position. We observed that the MmpL3’s secondary structure in H68A was also altered during binding with TMM (Fig. 7A). The large side chain of H68 in the WT MmpL3 creates a steric hindrance, which exposes the S66 and D58 residues more and helps stabilize the salt bridge between D139 and R63 (occupancy rate of 15.2%). Mutating H68 to alanine reduces the exposure of S66 and D58, nearly eliminating this salt bridge (occupancy rate decreases to 0.50%). This change allows R63 and D139 to form more H-bonds with TMM, positioning it deeper along the membrane normal (Fig. 7B). As TMM shifts further from its original binding position in WT (Fig. 7B), a notable change occurs: Y44 forms an H-bond with the hydroxyl group of TMM more frequently (Fig. 7B). This is evidenced by an occupancy rate of 27.8% for this interaction in the mutant, a substantial increase from the 5.8% observed in the WT. This enhanced interaction with Y44 contributes to the stable anchoring of TMM in the deeper position within the binding pocket.

**Fig 8:**
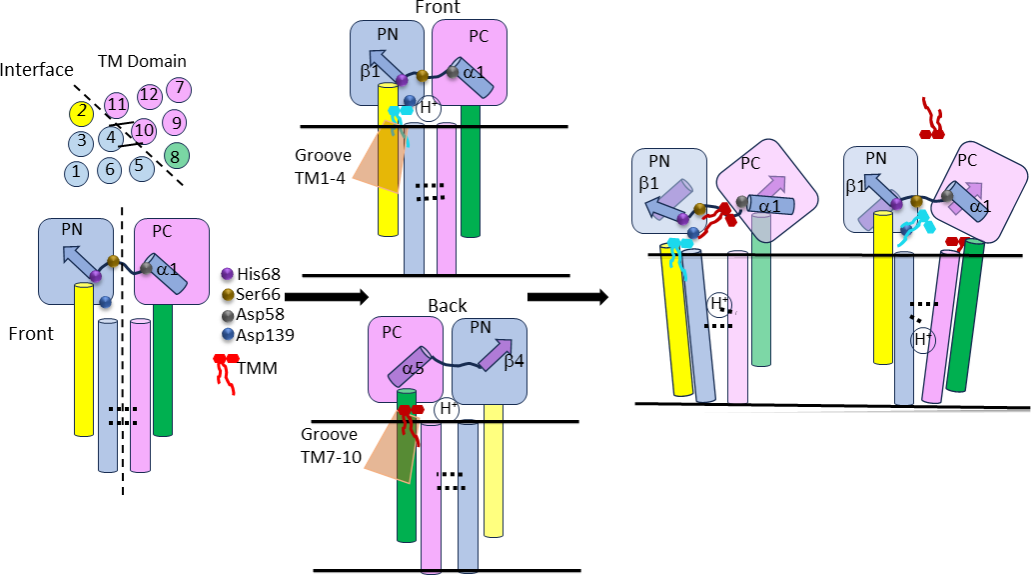
Proposed mechanism of MmpL3 transport of lipids. The H^+^ relay residues, Asp251, Tyr252, Asp640, and Tyr641, are located at the interface between the N-terminal (TM1-6) and C-terminal (TM7-12) TM bundles of MmpL3. The movements of TM2 and TM8 are coupled to the two loops in the PBP that serve as sensors of the ligands in the binding site. The conformational motions in the transporter are triggered by diffusion of a ligand from one of the two grooves into the PBP followed by the rearrangement of the H^+^-bond network of the I69-R63 loop connecting the α1 helix in the PC to the TM2 protruding into the PN. The downward sliding of TM2 enabled by disengagement from H68 could be transmitted to PC by the loop and the α1 helix. The H^+^ transfer is coupled to opening of the binding site into the periplasm and release of the substrate.

In summary, our simulations of the mutants demonstrate the significance of the five residues in the binding of MmpL3 substrates and highlight the distinct impacts that substitutions have on the position and interactions of TMM within the PBP. The simulations confirm that the hydroxyl group in TMM forms H-bonds with PBP residues, which aids in its deeper penetration into the binding pocket. These findings pave the way for future investigations, such as employing advanced constant-pH simulation methods (19, 20) to further explore how the mutations studied here may affect proton transport through the proton relay formed by the pair of dyads in the center of the TMs (Fig. 8).

## DISCUSSION

Recent progress in structural and functional analyses of MmpL3 dramatically advanced our understanding of the molecular mechanism of this subfamily of RND transporters. This study fills in an important gap in this understanding by reconstituting and characterizing the substrate transport activity of *Mtb* MmpL3 and identifying the mechanism of coupling the substrate translocation and energy provided by the downhill transfer of protons. We found that MmpL3 is optimized for the affinity and turnover of diverse substrates and that various ligands compete for the transporter. Importantly, we found that interactions of substrates within the periplasmic binding pocket are allosterically communicated to the proton relay in the transmembrane domain of MmpL3.

We analyzed both trans- and intermembrane lipid transport and their energy requirements. Our results show that the reconstituted *Mtb* MmpL3 promotes an asymmetric distribution of NBD-PE in the lipid bilayer with more of this ligand localized in the outer leaflet, the result suggesting a flippase-like activity (Fig. 6). This activity, however, does not require an input of energy and is not sensitive to the substitutions in the PBP, as seen from the lack of inhibition by SQ109 and independence from ΔpH (Fig. 6E). It is possible that the limitations of the reconstituted system, such as the phospholipid composition of the reconstituted proteoliposomes, which is different from the composition of the native *Mtb* cytoplasmic membrane, could affect the activity of the transporter. We also cannot exclude that MmpL3 might display flippase activity on TMM despite not being capable of flipping phospholipids. In addition, the C-terminal domain of MmpL3 and its interactions with other proteins could modulate the flippase activity of MmpL3. We note, however, that the C-terminal domain was found to be non-essential for cell growth (13, 17), and hence, protein activities associated with this domain, if any, are not required for the intracellular function of MmpL3.

The two TM grooves in MmpL3 formed by TMs1–4 or 7–10 of MmpL3, respectively, may contribute not only to diffusion of lipids from the outer leaflet into the periplasm but also to the flipping of lipids across the membrane. However, the extraction of fluorescently labeled PE lipids from the outer leaflet requires an input of energy and is inhibited not only by SQ109 but also by trehalose and Tre-C16 (Fig. 4), suggesting that all these ligands are transported by the same mechanism.

In agreement with structural data, we found that ligand binding in the PBP is important for the activity of MmpL3. Most of the ligand specificity comes from H-bonding of the lipid/detergent headgroups with specific amino acid residues in the PBP, which vary depending on the ligand. The D58A and D139A variants were efficient in PE and H^+^ transport assays but showed distinct interactions with trehalose/Tre-C16 ligands and SQ109 inhibitor. Structural analyses showed that D139 forms H-bonds with the trehalose moiety of TMM, but not with the headgroup of PE (9, 12). This residue is also H-bonded with Q40 and could contribute to controlling the access of ligands to the PBP from the intramembrane groove formed by TM1–4. The lack of competition between the PE lipids and trehalose/Tre-C16 in D139A variant further suggests that these ligands may use different grooves when entering the PBP, with Tre-C16/TMM entering from the TM7–10 groove as seen in structures whereas PE uses both grooves (Fig. 8). The MD simulations suggest that the D139A substitution eliminates H-bonding for all analyzed residues except S66 (Table 1), which leads to a different orientation of the trehalose headgroup in the PBP. Interactions with S66 appear to be important and sufficient for coupling with H^+^ relay sites as seen from the inhibition of D139A-mediated transport by SQ109 (Fig. 4F).

Likewise, in cryo-EM structures of MmpL3, D58 does not interact with PE but is H-bonded with TMM (9, 12). The PE transport mediated by the D58A variant is inhibited by trehalose/Tre-C16 but less efficiently than the WT protein (Fig. 4E). In particular, the IC_50_ of Tre-C16 increased from ~6.5 nM against the WT protein to ~83 nM against the D58A variant. In simulations of the D58A variant, the H-bonding network is also disrupted and redirected toward the H68 residue. As a result, TMM is positioned closer to the periplasmic exit, perhaps leading to the partial resistance of D58A to inhibition by SQ109.

The H68A and S66A mutant variants were both defective in the transport of lipids as well as H^+^ transfer (Fig. 3 and 4), whereas the neighboring R63A variant lost its structural integrity. This finding highlights the important role of the loop connecting the PN and PC domains in transport activity. Neither of these residues contacted the ligands in available structures of the ligand-bound MmpL3 proteins but appeared to H-bond with TMM to some extent according to MD simulations. In the *Mtb* MmpL3 structure (15), S66 residue is H-bonded with D54 in the loop and S131 in the PN shaping the PBP. In agreement, the S66A substitution demolished H-bonding with TMM in PBP, explaining the deep loss of the function of this transporter variant.

Interestingly, the backbone of H68 is H-bonded with the backbone of L166 in TM2, which extends all the way from the membrane and deep into PN. TM2 and TM8, the mirror of TM2 but inserted into the PC, are critical for transmitting conformational changes between the PBP and the H^+^ relay residues as well as for coupling of substrate and H^+^ transport reactions in homologous RND transporters, such as HAE-1 efflux pumps in Gram-negative bacteria, e.g., *E. coli* AcrB (21, 22). Specifically, TM2 is thought to move up and down perpendicular to the plane of the membrane, probably driving the rigid body rotation of the PC, which is also seen in MmpL3 (11).

Importantly, the H^+^ relay residues are located at the interface between the N-terminal (TM1–6) and C-terminal (TM7–12) TM bundles of MmpL3 (Fig. 8). We propose that in analogy with AcrB, the side-chain network formed by the two Asp-Tyr dyads in TM4 and TM10 of MmpL3 is rearranged in response to the movements of TM2 and TM8 allowing deeper water access and translocation of H^+^. The structure of MmpL3, like other RND transporters, contains two symmetric halves, each containing a channel that starts either at the TM1–4 groove or the TM7–10 groove (Fig. 8, indicated as front and back) leading into the PBP. Our results suggest that the conformational motions in the transporter are triggered by diffusion of a ligand from one of the two grooves into the PBP followed by the rearrangement of the H^+^-bond network of the I69-R63 loop connecting the α1 helix in the PC to the TM2 protruding into the PN (Fig. 8). The downward sliding of TM2 enabled by disengagement from H68 could be transmitted to PC by the loop and the α1 helix. The H^+^ transfer is coupled to the opening of the binding site into the periplasm and release of the substrate. A rigid body rotation of the PC domain with respect to PN was proposed previously to contribute to the extrusion of substrates by MmpL3 into the periplasm (11).

It remains unclear whether both halves of MmpL3 undergo similar conformational motions as was suggested for AcrB (21) or only the PC. AcrB is an obligate trimer, in which during transport each protomer assumes one of the three conformational states corresponding to different positions of a ligand in the PBP: access, binding, and extrusion (21, 23, 24). In contrast, MmpL3 oligomerization is not required for transport activities, and each protomer functions independently (10). Therefore, both symmetric and asymmetric conformational motions of the two halves are possible. The two ligand sensors, the I69-R63 loop connected to the α1 helix and the N450-P445 loop connected to the α5 helix, share certain features, with both containing Arg residues R63/R65 and R448, respectively, and hydroxyl-containing amino acids S66 and T449, respectively. The D58 of the α1 helix is matched by D441 in the α5 helix, whereas H68 bonding with TM2 is mirrored by N450 with TM8. Hence, both loops could communicate the conformational changes in PN and PC domains to the H^+^ relay residues through TM2 and TM8, respectively. Our results show that despite significant loss in their transport activities, the H68A and S66A variants still enable bacterial growth (Fig. 2), suggesting that MmpL3 lacking this sensor can translocate TMM, albeit less efficiently.

## MATERIALS AND METHODS

### Bacterial strains and growth conditions

*M. smegmatis* mc^2^155 was grown in Middlebrook 7H9 broth (Difco) with 10% albumin–dextrose–catalase (ADC) supplement and 0.05% Tween 80 and on 7H11 agar plates containing 10% ADC supplement at 37°C. Where required, kanamycin (Kan) (25 mg/L), hygromycin (Hyg) (50 mg/L), and sucrose (10%) were added to the culture medium.

### Msmg recombinant strains expressing mmpL3 mutated variants

Mutated variants of *mmpL3* gene from *Mtb* were generated by site-directed mutagenesis (GenScript, Piscataway, NJ, USA). The activity of the different point-mutated MmpL3 variants was determined by testing their ability to rescue the growth of a *Msmg* mmpL3 knock-out mutant (*Msmg*Δ*mmpL3*) with the entire *mmpL3* coding sequence deleted by allelic replacement. Briefly, replicative pMVGH1 plasmid constructs expressing different *mmpL3* mutants (MmpL3^D64A^, MmpL3^S66A^, MmpL3^H68A^, MmpL3^Q40C^, MmpL3^D58A^, and MmpL3^D139A^) under control of the phsp60 promoter were used to transform a *Msmg* strain having undergone a single crossover event at its *mmpL3* locus as described (17), and the resulting transformants were next plated on 7H11-ADC agar containing Kan, Hyg, and sucrose to select for allelic exchange mutants. The production of MmpL3 proteins by these mutants was analyzed by immunoblot using a polyclonal rabbit antibody directed against the first periplasmic loop of MmpL3 from *M. tuberculosis* as described (8).

### Whole-cell radiolabeling experiments

Metabolic labeling of whole *Msmg* cells with [1,2-^14^C]acetic acid (0.5 mCi/mL; specific activity, 52 Ci/mol, Perkin Elmer) was performed in 7H9-ADC-Tween 80 medium for 4 h at 37°C with shaking.

### Analytical procedures

Total lipid extraction from bacterial cells and preparation of fatty acid and mycolic acid methyl esters from extractable lipids and delipidated cells followed earlier procedures (4). [1,2-^14^C]acetic acid-derived lipids and fatty acid/mycolic acid methyl esters were separated by TLC on aluminum-backed silica gel 60-precoated plates F_254_ (E. Merck) and imaged using a Sapphire Biomolecular Imager (Azure Biosystems).

### Protein purification

The DNA sequences encoding the N-terminal 767 residues of the constructed *Mtb* MmpL3 and its mutated variants (MmpL3ΔC) were subcloned into the pET21a expression vector in frame with a 6×His tag at the C-terminus and under the control of the isopropyl-β-D-thiogalactopyranosid (IPTG)-inducible T7 promoter. The resultant plasmids were transformed into *E. coli* Rosetta (DH3) cells by chemical transformation.

Ten milliliters of overnight cell culture grown in Luria–Bertani broth supplemented with 100 µg/mL ampicillin was added to 1 L of the fresh medium and incubated at 37°C until the optical density at 600 nm (OD_600_) reached 0.5. Then, the growing cells were treated with 0.1 mM IPTG to induce the expression of *mmpL3*Δ*C* and its mutated variants. After 3 h of induction, cells were harvested by centrifugation at 4,200 × *g* for 1 h at 4°C and resuspended in ice-cold low-salt buffer (100 mM sodium phosphate [pH 7.2], 10% glycerol, 1 mM ethylenediaminetetraacetic acid [EDTA], and 1 mM phenylmethanesulfonyl fluoride [PMSF]). The cell suspension was then passed three times through the French press. Insoluble material was removed by centrifugation at 4,200 × *g* for 1 h at 4°C. The membrane fraction was collected by ultracentrifugation and washed twice with high-salt buffer (20 mM sodium phosphate [pH 7.2], 2 M KCl, 10% glycerol, 1 mM EDTA, and 1 mM PMSF) as described before (9).

The MmpL3ΔC and its mutated variants were solubilized in 10% (wt/vol) HS buffer (50 mM HEPES [pH 7.0], 150 mM NaCl, 2% DDM, and 1 mM PMSF) at 4°C by overnight stirring, and the insoluble material was removed by ultracentrifugation at 100,000 × *g* for 1 h at 4°C. The extracted sample was then purified using 1 mL HiTrap (GE Healthcare) Cu^2+^-affinity column equilibrated in HS buffer with adjusted concentration of NaCl to 400 mM and imidazole to 20 mM. The column was washed with 40 CVs of HW20 buffer (50 mM HEPES [pH 7.0], 400 mM NaCl, 0.2% DDM, and 20 mM imidazole) and 2 CVs of HW50 buffer (50 mM HEPES [pH 7.0], 400 mM NaCl, 0.2% DDM, and 50 mM imidazole). MmpL3ΔC and its variants were eluted with HW500 buffer (50 mM HEPES [pH 7.0], 400 mM NaCl, 0.2% DDM, and 500 mM imidazole). The purity of proteins was confirmed using SDS-PAGE stained with Coomassie Brilliant Blue. Protein samples were stored at 4°C for reconstitution into liposomes for up to 3 days.

### Reconstitution of MmpL3ΔC into proteoliposomes

*E. coli* polar lipids (Avanti) were dissolved in chloroform and dried under vacuum overnight. Prior to reconstitution, lipids were resuspended in reconstitution buffer (25 mM HEPES-KOH [pH 7.0], 100 mM KCl) to a concentration of 20 mg/mL and sonicated briefly (Branson 1510 water-bath sonicator). One hundred twenty-five microliters of dispersed lipid solution was mixed with 10, 25, or 50 µL of the protein sample and supplemented, and the volume was adjusted to 500 µL via the addition of reconstitution buffer supplemented with 1.1% n-octyl-β-D-glucopyranoside.

The sample was water bath-sonicated for 1 min, then diluted slowly to 3 mL with reconstitution buffer, and allowed to dialyze for 2 h at 4°C with stirring against reconstitution buffer supplemented with SM-2 BioBeads (2 g/L). The sample was then ultracentrifuged at 100,000 × *g* for 60 min, and the resulting pellet was resuspended in 200 µL of reconstitution buffer. Pyranine was added to the sample at a final concentration of 2 mM, and the sample was extruded through a 200-nm pore (Avanti Mini Extruder). The sample was then passed through Illustra NAP-5 Columns (GE Life Sciences) to remove untrapped pyranine. Control vesicles devoid of Mmpl3_767_ were prepared in parallel by the addition of reconstitution buffer instead of protein (10).

### Reconstitution of MmpL3ΔC into fluorescent proteoliposomes

For the preparation of fluorescent donor proteoliposomes and lipid vesicles, 0.4 mL *E. coli* polar lipid solution in chloroform (25 mg/mL) was mixed with 1,2-dipalmitoyl N-(7-nitrobenz-2-oxa-1,3-diazol-4-yl)phosphatidylethanolamine (16:0 N-NBD-PE, Avanti) and 1,2-dipalmitoyl N-(lissamine rhodamine B sulfonyl)phosphatidylethanolamine (16:0 N-Rh-PE, Avanti) in a 99:0.5:0.5 molar ratio. The mixture was dried down in a glass tube under nitrogen, and the tubes were kept under reduced pressure overnight. The lipid was rehydrated in the reconstitution buffer at room temperature, and then, proteoliposomes were prepared as described above. The proteoliposome suspension after extrusion was adjusted to 4 mL with reconstitution buffer (16).

Unlabeled acceptor lipid vesicles were prepared using the same protocol but without adding fluorescent PE derivatives. The sizing of reconstituted vesicles was done by extrusion of samples through a membrane with 200-nm pores (Avanti Mini Extruder) (16).

### Quantification of proteins reconstituted into proteoliposomes and protein–lipid polar ratio in liposomes

The protein concentration in proteoliposomes was determined using a standard protocol of SDS-PAGE and staining the gels with Coomassie Brilliant Blue (Fig. S2). The phospholipid concentration was determined using the total phosphorus assay (25). Three hundred microliters of liposome sample was mixed with 100 µL of 10% ascorbic acid and 600 µL of 0.42% ammonium molybdate⋅4 H_2_O and incubated for 1 h at 37°C. A blank sample was prepared the same way with deionized water instead of liposomes. The optical density was measured against a blank sample at 820 nm on a spectrometer (ThermoFisher Scientific, Genesis30).

Typically, the final amount of total phospholipids in 50 µL of the donor MmpL3ΔC variants-containing proteoliposomes was 60–70 nmol with the protein:lipid ratio ~6–11:1,000.

### Transmembrane proton flux assay

All experiments with proteoliposomes were carried out in reconstitution buffer (25 mM HEPES-KOH [pH 7.0] with 100 mM KCl). For the experiments with ΔpH, the reconstitution buffer was adjusted to pH 6.0 and pH 8.0. For proton transport assays, experimental compounds were mixed with 100 µL of vesicles, and then, 10 µL of premixture was added to 90 µL of the reconstitution buffer. Data collection began immediately on a 96-well plate. Fluorescence measurements were carried out with a Tecan Spark 10M microplate reader at room temperature. The excitation and emission wavelengths used for pyranine were 455 and 509 nm, respectively.

### Intermembrane transport of fluorescent phospholipids

MmpL3-dependent extrusion of fluorescent phospholipids was measured by quenching of NBD fluorescence due to a FRET to N-Rh-PE. Fluorescence measurements were carried out after 3 seconds of shaking the plate holder at 30°C in a Tecan Spark 10M microplate reader. Proteoliposome suspensions were loaded into an injector. In all experiments, 50 µL of proteoliposomes was added to 150 µL of experimental mixture that contained 146 µL of buffer and 4 µL of acceptor liposomes. The excitation and emission wavelengths used for N-NBD-PE were 460 and 538 nm, respectively.

The reaction was initiated by the addition of MgCl_2_ with a final concentration of 5 mM, and the increase of NBD fluorescence was followed. After the termination of the reaction by the addition of EDTA (final concentration 10 mM), the N-NBD-PE fluorescence was completely dequenched by solubilizing vesicles with the detergent dodecyl maltoside (final concentration of 0.5%) (Fig. S8). Protein-free donor vesicles prepared along with proteoliposomes were used as a control.

### Phospholipid flippase activity assay

For the preparation of NBD-proteoliposomes and lipid vesicles, *E. coli* polar lipid solution in chloroform was mixed with 1,2-dipalmitoyl N-(7-nitrobenz-2-oxa-1,3-diazol-4-yl)phosphatidylethanolamine (16:0 N-NBD-PE, Avanti) in a 99:1 molar ratio (26). MmpL3 flippase activity was measured by DTN quenching of NBD fluorescence in the outer leaflet of the proteoliposomes. Proteoliposome suspension (10 µL) was added to 190 µL of buffer. Fluorescence measurements were carried out at different external pH values (25 mM MES-KOH with 100 mM KCl [pH 6.0] or 25 mM HEPES-KOH with 100 mM KCl [pH 7.0 or pH 8.0]) at 30°C in a Tecan Spark 10M microplate reader (λ_ex_ = 460 nm and λ_em_ = 536 nm) and monitored for 3.5 min. The quenching reaction was initiated after 20 seconds with the addition of 2 µL of 1 M DNT in 0.5 M Tris-HCl buffer (pH 9.5) (freshly prepared and kept on ice) to the final concentration of 10 mM. The fluorescence was monitored until a stable line was observed. After quenching the fluorescence of NBD-phospholipids in the outer leaflet of the vesicles by DTN, the reaction was stopped by adding Triton X-100 detergent (final concentration of 0.5%), which solubilized all vesicles leading to the exposure of the remaining inner leaflet NBD-lipids to DTN. Protein-free donor vesicles prepared along with proteoliposomes were used as a control.

### MD simulation

The simulation systems were constructed based on a previous study (14), utilizing the *Mtb* MmpL3 structure (PDB:7NVH) and a symmetric model of the mycobacterial inner membrane with the following lipids: 1-tuberculostearoyl-2-palmitoyl-sn-glycero-3-phosphorylethanolamine (TPPE), cardiolipin (10,30-bis[1-oleoyl-2-palmitoleoyl-sn-glycero-3-phospho]-glycerol), 1-palmitoyl-2-oleoyl-sn-glycero-3-phosphatidylinositol (POPI), and four varieties of acylated phosphatidylinositol mannosides, AcPIM_2_, Ac_2_PIM_2_, AcPIM_6_, and Ac_2_PIM_6_. The total number of each membrane component was set to TPPE, 88; cardiolipin, 118; POPI, 6; AcPIM_2_, 35; Ac_2_PIM_2_, 35; AcPIM_6_, 17; and Ac_2_PIM_6_, 17. All residues of MmpL3 were assigned default protonation states, namely, protonated for lysines and arginines and deprotonated for glutamates and aspartates. Mutations were introduced using VMD (27). The system was solvated with TIP3P water (28) above and below the protein complex. Sodium (Na^+^) and chloride (Cl^−^) ions were added to the bulk water to achieve a concentration of 150 mM. The final system size was approximately 250,000 atoms.

Simulations were conducted using NAMD3 (29), employing the CHARMM36m force field for proteins (30) and CHARMM36 for lipids (31). Hydrogen mass repartitioning (32) was implemented to facilitate a uniform time step of 4 fs. The simulations were maintained at a constant temperature of 310 K and a pressure of 1 atm using Langevin dynamics and a Langevin piston, respectively (33, 34). As a starting point, we adopted the equilibrated system from our previous study (14), for which a multistep relax protocol was applied. In the first step, all components except the lipid acyl chains were constrained for 1 ns, allowing them to become fluid. Following this, we released all system components except for the protein for 10 ns. Next, the protein sidechains were released, and an additional 10 ns was run. Subsequently, the entire system was released for a 100-ns equilibration. After ensuring the protein and membrane are relaxed, we introduced a TMM molecule into the periplasmic cavity and created the five mutated systems, D139A, D58A, H68A, R63A, and S66A, in addition to the WT system. The resulting final systems were then minimized for 2,000 steps and used for production runs. We ran simulations for both the WT (with three replicas) and five mutants (D139A, D58A, H68A, R63A, and S66A), each with TMM bound, for a duration of 1 μs each. This resulted in a cumulative simulation time of 8 μs. Analysis of hydrogen bonds was carried out with the HBonds plugin in VMD using the last 800 ns of each simulation. For the WT system, the reported hydrogen bonds are the average of the three replicas. RMSD was calculated for each system relative to its starting state, excluding the unstructured cytoplasmic loop (residues 340–378).

## SUPPLEMENTAL MATERIAL

Fig. S1 mbio.02183-24-s0001.tif Periplasmic ligands.

Fig. S2 mbio.02183-24-s0002.tif Protein and lipid quantification in reconstituted fluorescent proteoliposomes.

Fig. S3 mbio.02183-24-s0003.tif Proton transfer activity.

Fig. S4 mbio.02183-24-s0004.tif Intermembrane lipid transport activity.

Fig. S5 mbio.02183-24-s0005.tif The rates of intermembrane lipid transport.

Fig. S6 mbio.02183-24-s0006.tif Molecular dynamics simulations.

Fig. S7 mbio.02183-24-s0007.tif Histograms of the distances.

Fig. S8 mbio.02183-24-s0008.tif Fluorescence controls.

Table S1 mbio.02183-24-s0009.docx Proton transfer activity of MmpL3 variants.

Table S2 mbio.02183-24-s0010.docx Lipid transport activity of MmpL3 variants.

ASM does not own the copyrights to Supplemental Material that may be linked to, or accessed through, an article. The authors have granted ASM a non-exclusive, world-wide license to publish the Supplemental Material files. Please contact the corresponding author directly for reuse.
