# Supplementary figures and images for: Allosteric coupling of substrate binding and proton translocation in MmpL3 transporter from Mycobacterium tuberculosis
Source: mBio. 2024 Aug 30;15(10):e02183-24. doi: 10.1128/mbio.02183-24 (PMC11481577; doi:10.1128/mbio.02183-24)

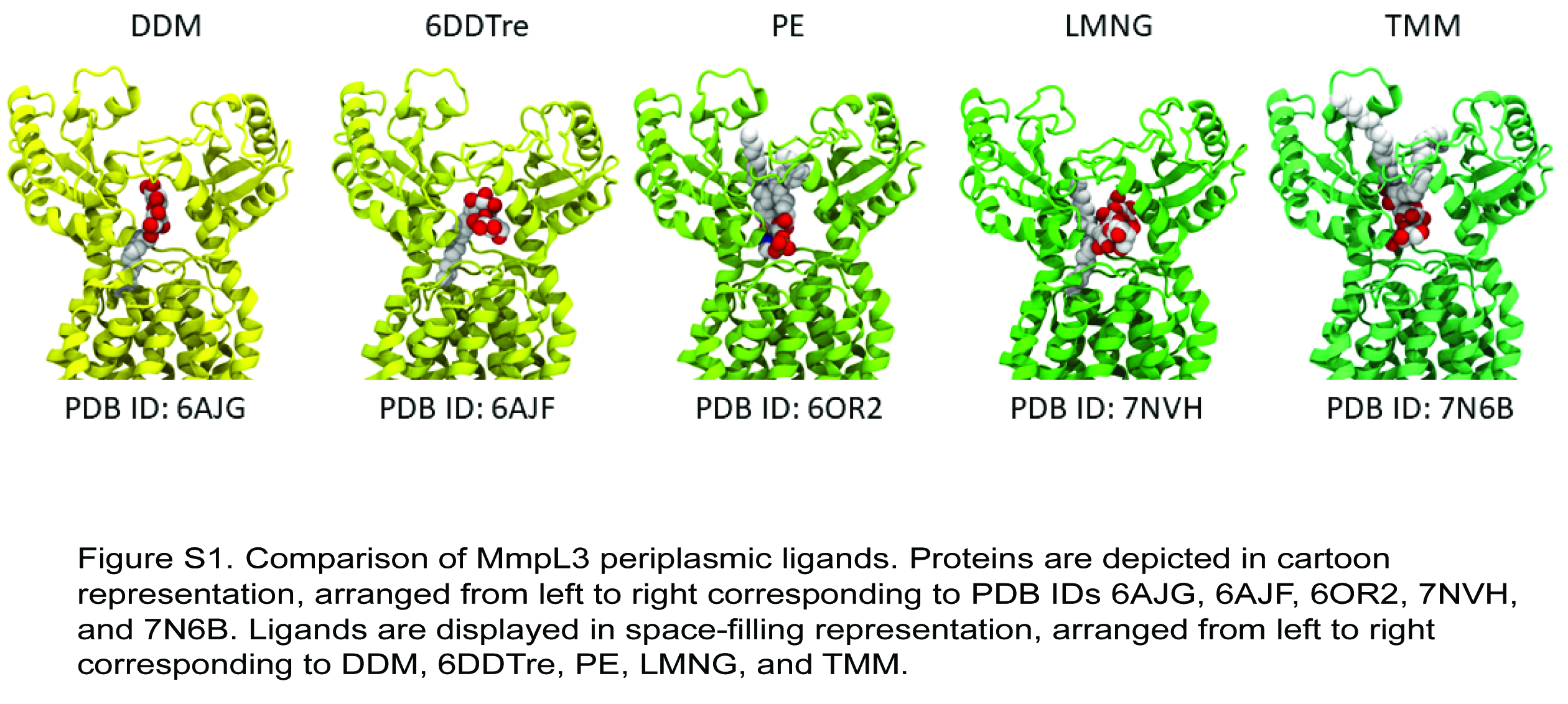

Supplement: Fig. S1 — Periplasmic ligands. [file mbio.02183-24-s0001.tif]

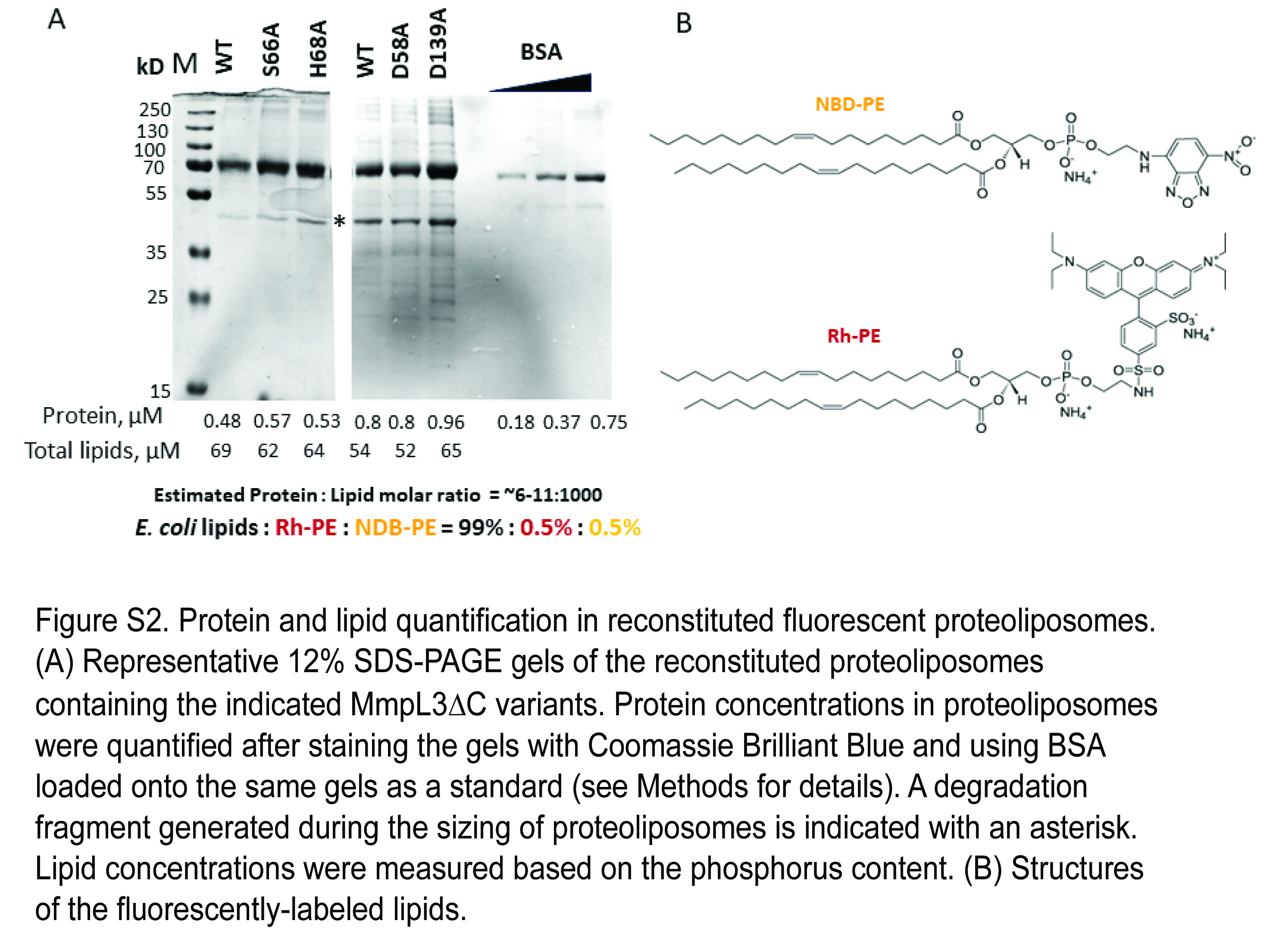

Supplement: Fig. S2 — Protein and lipid quantification in reconstituted fluorescent proteoliposomes. [file mbio.02183-24-s0002.tif]

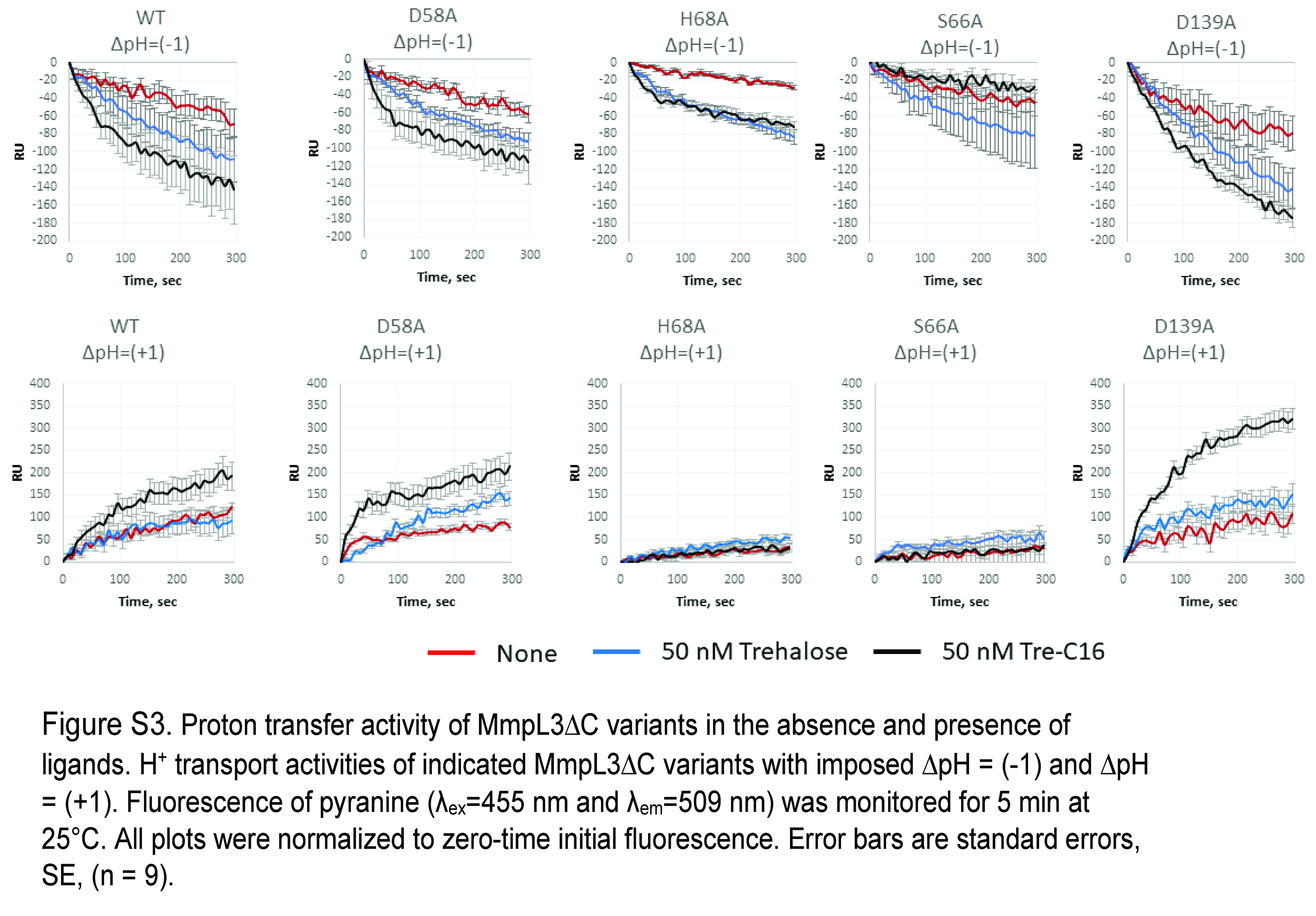

Supplement: Fig. S3 — Proton transfer activity. [file mbio.02183-24-s0003.tif]

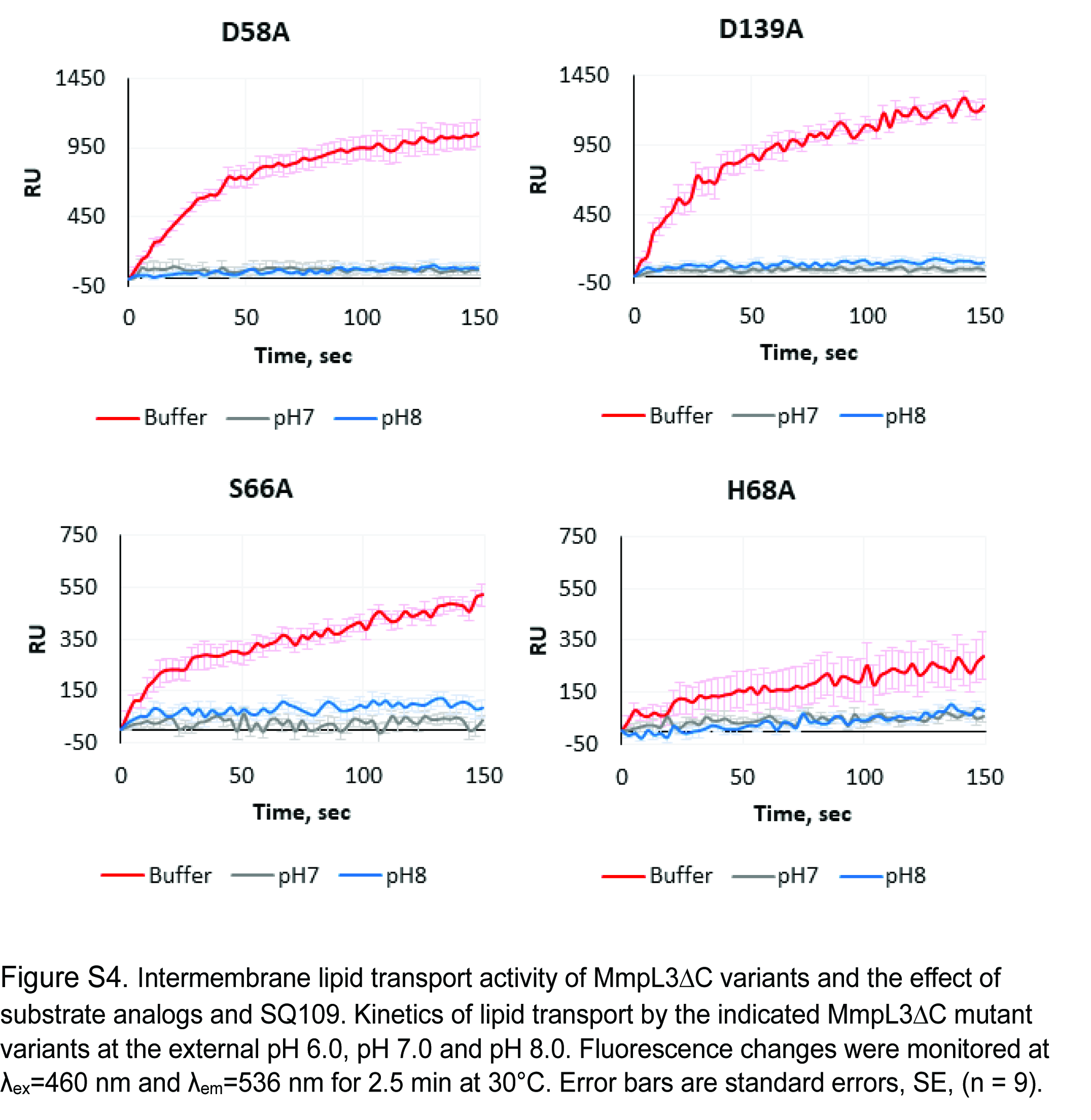

Supplement: Fig. S4 — Intermembrane lipid transport activity. [file mbio.02183-24-s0004.tif]

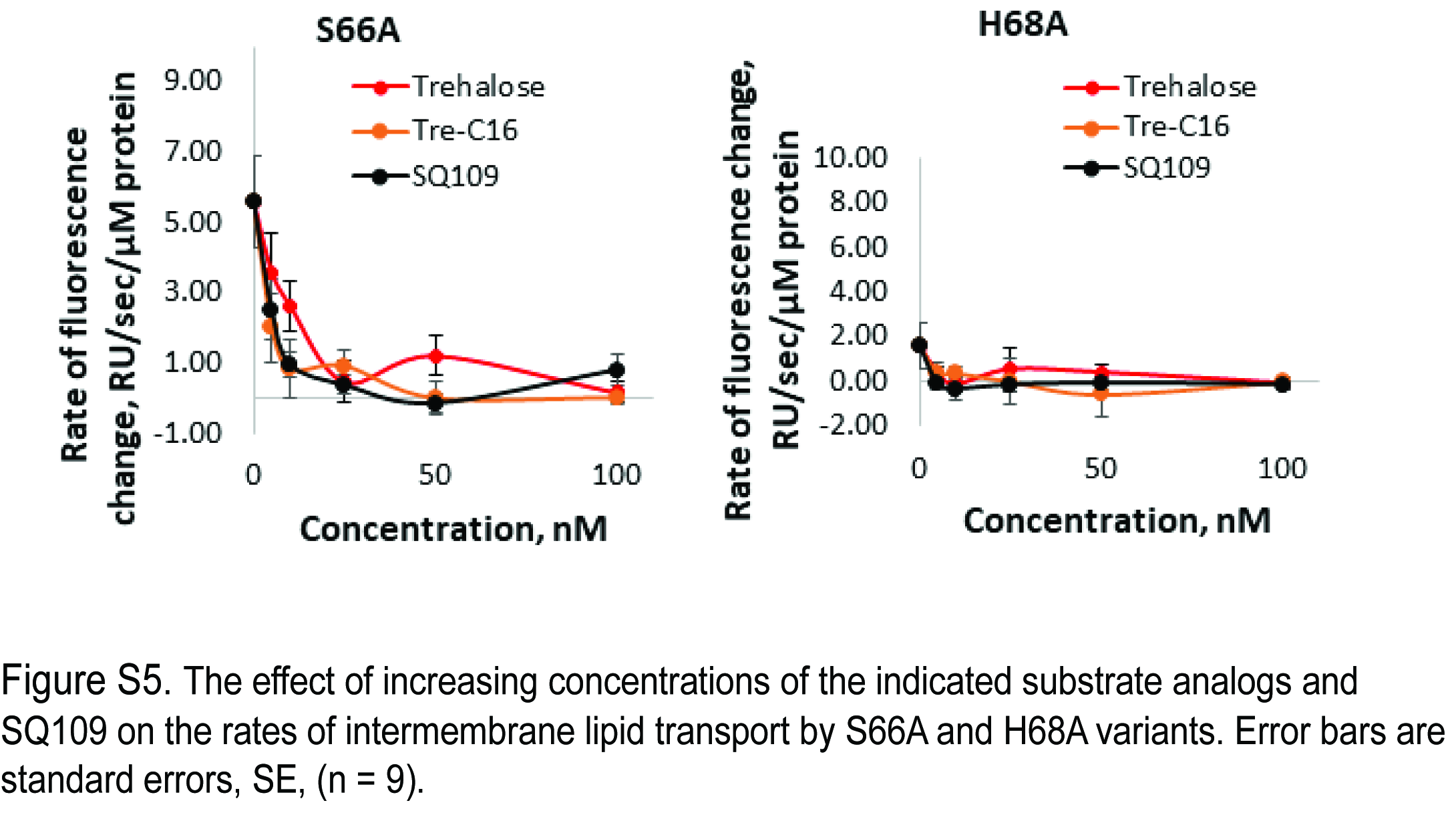

Supplement: Fig. S5 — The rates of intermembrane lipid transport. [file mbio.02183-24-s0005.tif]

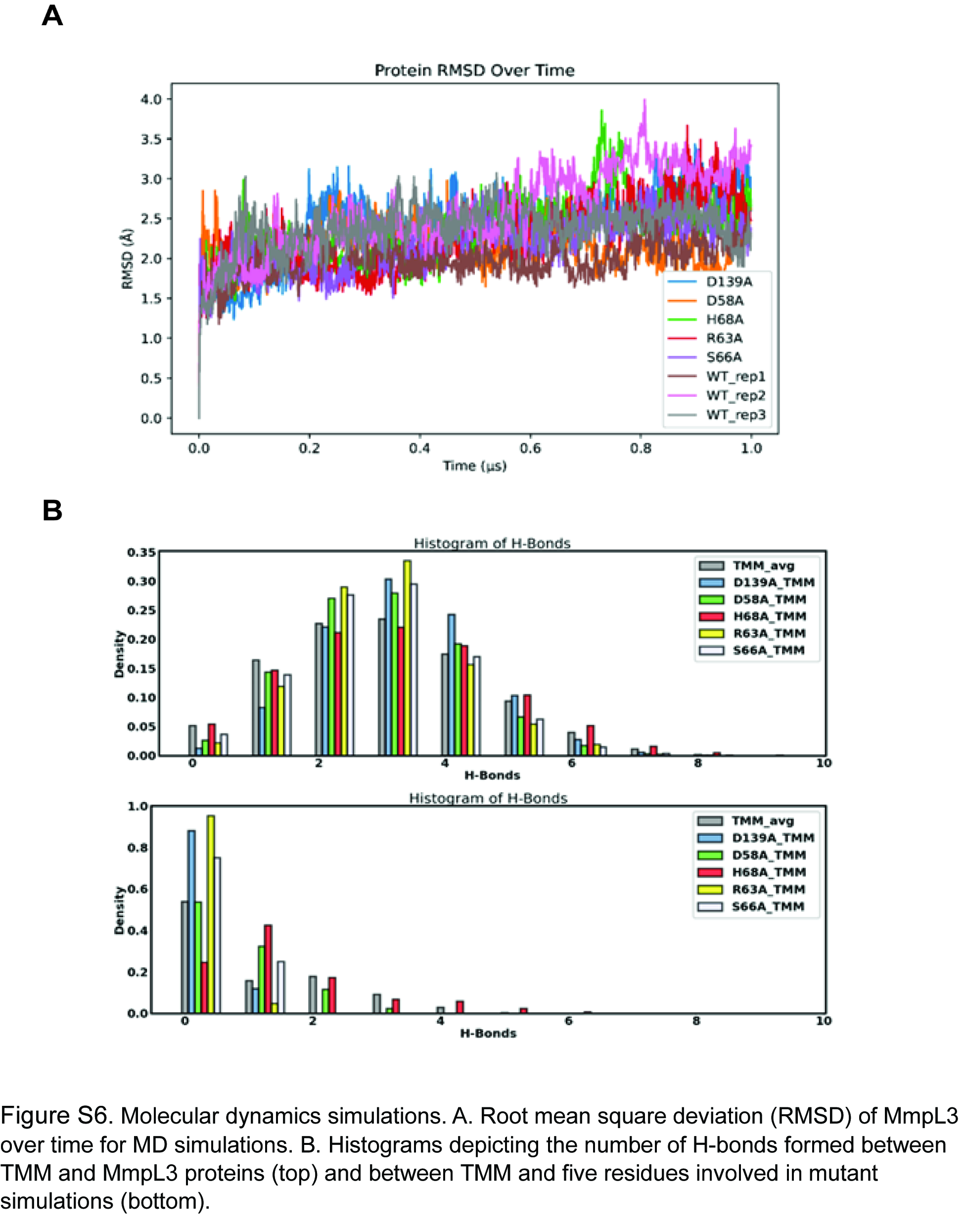

Supplement: Fig. S6 — Molecular dynamics simulations. [file mbio.02183-24-s0006.tif]

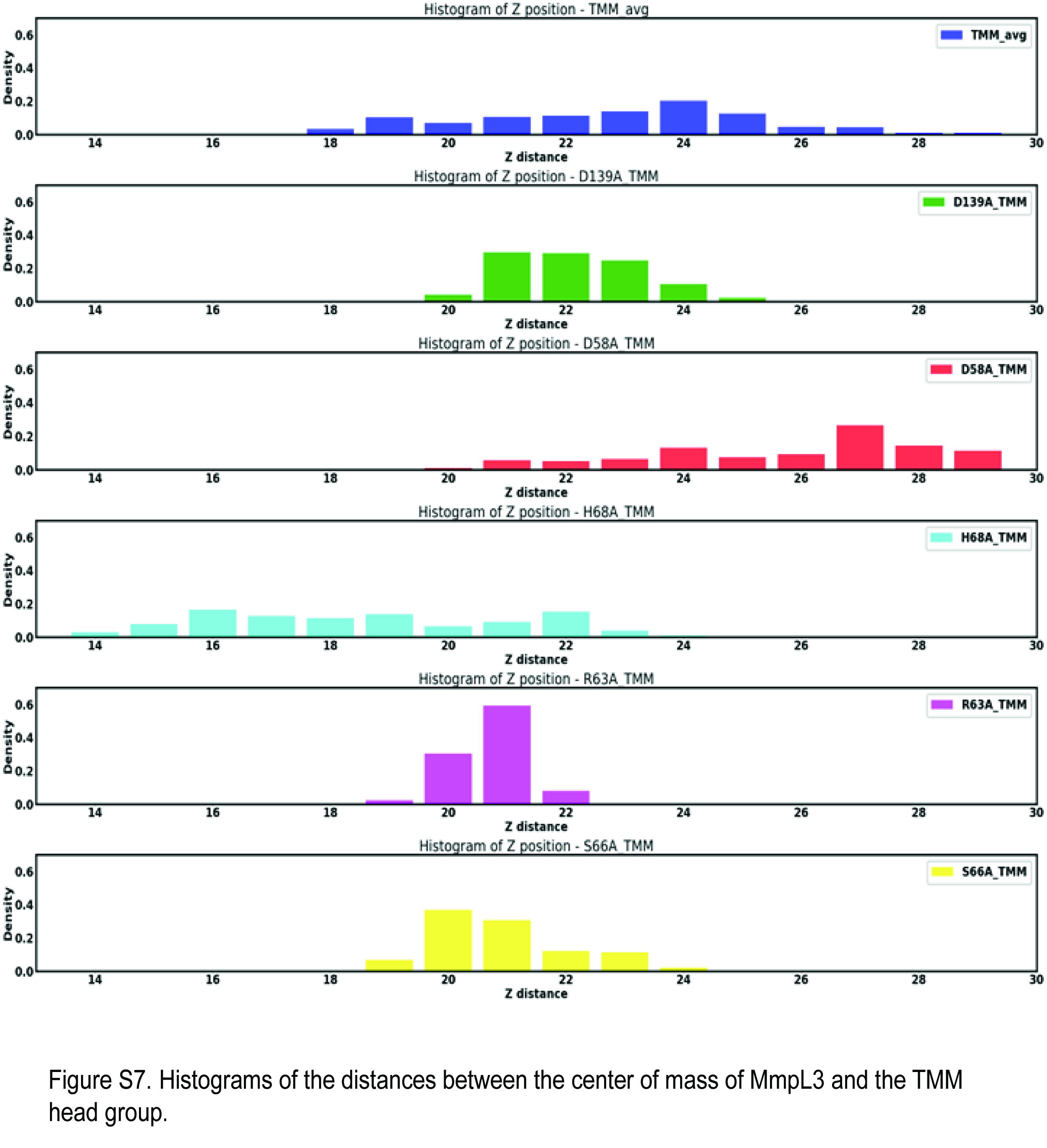

Supplement: Fig. S7 — Histograms of the distances. [file mbio.02183-24-s0007.tif]

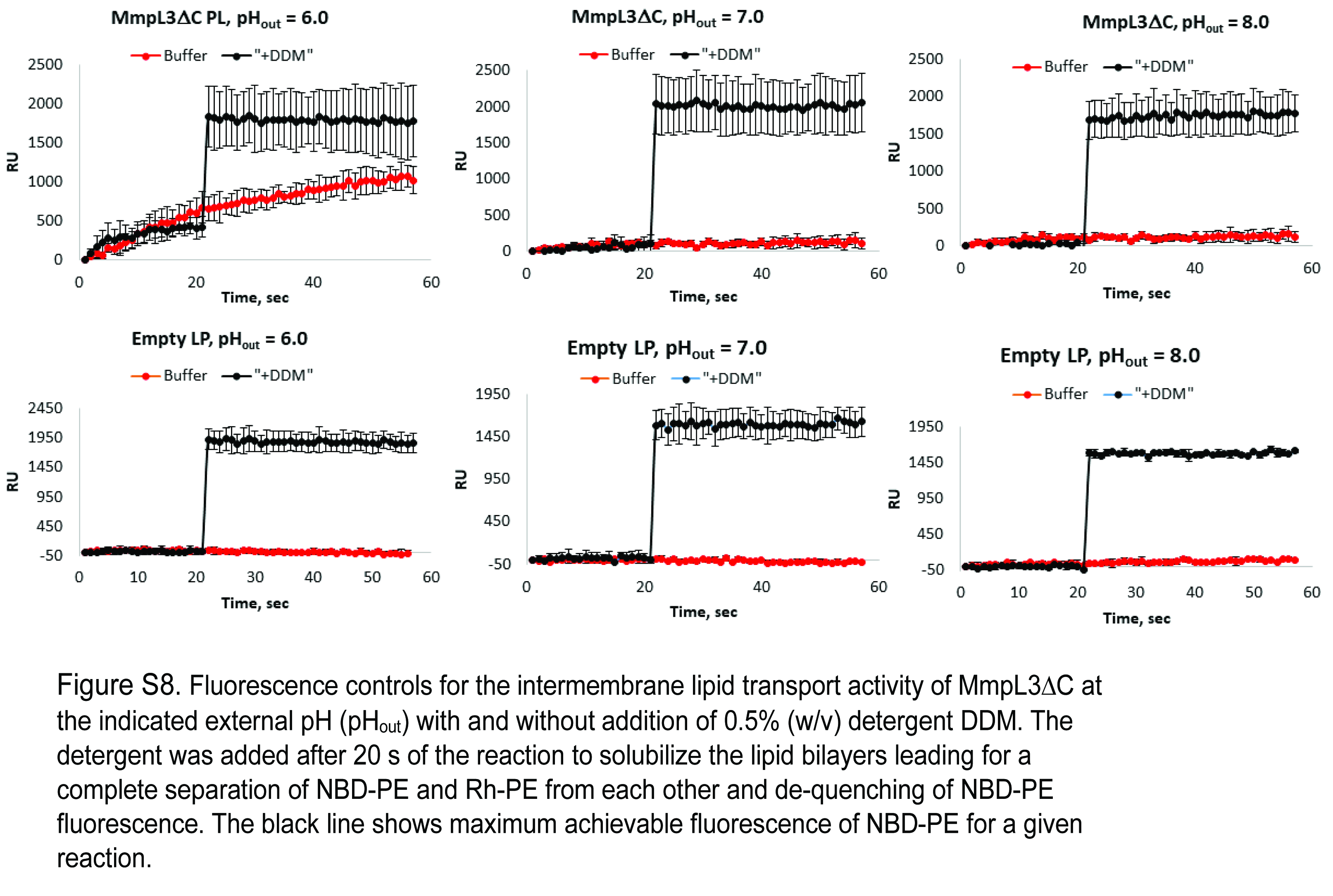

Supplement: Fig. S8 — Fluorescence controls. [file mbio.02183-24-s0008.tif]
