# Supplementary material for: Allosteric coupling of substrate binding and proton translocation in MmpL3 transporter from Mycobacterium tuberculosis
Source: mBio. 2024 Aug 30;15(10):e02183-24. doi: 10.1128/mbio.02183-24 (PMC11481577; doi:10.1128/mbio.02183-24)
Supplement: Table S1 — Proton transfer activity of MmpL3 variants. [file mbio.02183-24-s0009.docx]

Table S1. Proton transfer activity of MmpL3 variants.

|  | Area Under Curve (AUC), RU/μM protein | | | | | | | | | | | |
| --- | --- | --- | --- | --- | --- | --- | --- | --- | --- | --- | --- | --- |
|  | No additions | | | | | | AcTre67 (50 nM) | | | | | |
| Protein | ∆pH=(-1) | | | ∆pH=(+1) | | | ∆pH=(-1) | | | ∆pH=(+1) | | |
| WT | -0.14 | ± | 0.02 | 0.30 | ± | 0.02 | -0.27 | ± | 0.09 | 0.24 | ± | 0.10 |
| D58A | -0.11 | ± | 0.02 | 0.22 | ± | 0.02 | -0.21 | ± | 0.03 | 0.24 | ± | 0.04 |
| S66A | -0.11 | ± | 0.03 | 0.16 | ± | 0.03 | -0.13 | ± | 0.07 | 0.08 | ± | 0.07 |
| H68A | -0.06 | ± | 0.01 | 0.08 | ± | 0.02 | -0.21 | ± | 0.03 | 0.12 | ± | 0.03 |
| D139A | -0.94 | ± | 0.32 | 0.17 | ± | 0.33 | -1.33 | ± | 0.28 | 0.33 | ± | 0.29 |
