# Supplementary material for: Allosteric coupling of substrate binding and proton translocation in MmpL3 transporter from Mycobacterium tuberculosis
Source: mBio. 2024 Aug 30;15(10):e02183-24. doi: 10.1128/mbio.02183-24 (PMC11481577; doi:10.1128/mbio.02183-24)
Supplement: Table S2 — Lipid transport activity of MmpL3 variants. [file mbio.02183-24-s0010.docx]

Table S2. Lipid transport activity of MmpL3 variants expressed as rates of fluorescence change, RU/s/μM protein.

|  | Rate of fluorescence change, RU/s/μM protein | | | | | | | | | | | |
| --- | --- | --- | --- | --- | --- | --- | --- | --- | --- | --- | --- | --- |
|  | DMSO | | | Tre (50 nM) | | | Tre-C16 (50 nM) | | | SQ109 (50 nM) | | |
| Protein | ∆pH=(-1) | | | ∆pH=(-1) | | | ∆pH=(-1) | | | ∆pH=(-1) | | |
| WT | 24.14 | ± | 1.68 | 2.96 | ± | 1.07 | 1.32 | ± | 0.33 | 2.04 | ± | 0.90 |
| D58A | 19.39 | ± | 1.46 | 7.22 | ± | 1.49 | 11.53 | ± | 1.83 | 10.7 | ± | 2.24 |
| S66A | 5.59 | ± | 1.30 | 1.20 | ± | 0.56 | -0.02 | ± | 0.46 | -0.15 | ± | 0.25 |
| H68A | 1.58 | ± | 1.05 | 0.37 | ± | 0.36 | -0.62 | ± | 1.04 | -0.12 | ± | 0.32 |
| D139A | 16.71 | ± | 1.73 | 18.84 | ± | 1.38 | 13.43 | ± | 1.15 | 2.52 | ± | 0.93 |
